# Effect of Two Kinds of Fertilizers on Growth and Rhizosphere Soil Properties of Bayberry with Decline Disease

**DOI:** 10.3390/plants10112386

**Published:** 2021-11-05

**Authors:** Haiying Ren, Hongyan Wang, Zheping Yu, Shuwen Zhang, Xingjiang Qi, Li Sun, Zhenshuo Wang, Muchen Zhang, Temoor Ahmed, Bin Li

**Affiliations:** 1 Institute of Horticulture, Zhejiang Academy of Agricultural Sciences, Hangzhou 310021, China; renhy@zaas.ac.cn (H.R.); hongywang1@gmail.com (H.W.); yuzp@zaas.ac.cn (Z.Y.); zhangsw@zaas.ac.cn (S.Z.); qixj@zaas.ac.cn (X.Q.); lsun@zaas.ac.cn (L.S.); 2 College of Plant Protection, China Agricultural University, Beijing 100193, China; 3 Institute of Biotechnology, Zhejiang University, Hangzhou 310058, China; 11816060@zju.edu.cn (M.Z.); temoorahmed@zju.edu.cn (T.A.)

**Keywords:** bayberry, decline disease, compound fertilizer, bio-organic fertilizer, microbial community, metabonomics

## Abstract

Decline disease causes severe damage to bayberry. However, the cause of this disease remains unclear. Interestingly, our previous studies found that the disease severity is related with the level of soil fertilizer. This study aims to explore the effect and mechanism of compound fertilizer (CF) and bio-organic fertilizer (OF) in this disease by investigating the vegetative growth, fruit characters, soil property, rhizosphere microflora and metabolites. Results indicated that compared with the disease control, CF and OF exhibited differential effect in plant healthy and soil quality, together with the increase in relative abundance of *Burkholderia* and *Mortierella*, and the reduction in that of *Rhizomicrobium* and *Acidibacter*, *Trichoderma*, and *Cladophialophora* reduced. The relative abundance of *Geminibasidium* were increased by CF (251.79%) but reduced by OF (13.99%). In general, the composition of bacterial and fungal communities in rhizosphere soil was affected significantly at genus level by exchangeable calcium, available phosphorus, and exchangeable magnesium, while the former two variables had a greater influence in bacterial communities than fungal communities. Analysis of GC-MS metabonomics indicated that compared to the disease control, CF and OF significantly changed the contents of 31 and 45 metabolites, respectively, while both fertilizers changed C5-branched dibasic acid, galactose, and pyrimidine metabolic pathway. Furthermore, a significant correlation was observed at the phylum, order and genus levels between microbial groups and secondary metabolites of bayberry rhizosphere soil. In summary, the results provide a new way for rejuvenation of this diseased bayberry trees.

## 1. Introduction

Bayberry (*Myrica rubra*) is an economically important fruit tree and medicinal plant that is grown in southern China [1,2,3]. It is cultivated in about 334,000 hectares each year with an annual output of 950,000 tons. However, in recent years, the bayberry decline disease occurred seriously which led to sprout inhibition, photosynthetic rate reduction, soil quality degradation, and trees dying 3–4 years later [4]. The cause of this disease remains unclear. Interestingly, our previous studies found that the disease severity is associated with the level of soil fertilizer. Therefore, the development of effective rejuvenation technology and understanding of its mechanism will provide effective support for the healthy development of the bayberry industry. Interestingly, our previous studies found that the disease severity seems to be related with rhizosphere soil fertilization [4].

Fertilization is an effective measure to increase crop yield in agricultural production. In general, compound fertilizer and bio-organic fertilizer are often used in agriculture. Compound fertilizer refers to chemical fertilizer containing two or more nutrient elements with a high nutritional content that promotes the yield of grain, vegetables and fruits. However, excessive use of chemical fertilizer will lead to serious soil hardening and acidification, resulting in poor plant growth [5]. Bio-organic fertilizer is composed of specific functional microorganisms, decomposed organic fertilizer or animal residues, which can improve soil physical and chemical properties [6], enrich organic matter and balance nutrient levels [7], regulate the structure and function of the microbial community [8,9,10], promote plant growth, increase crop yield [11] and improve fruit quality [12]. The role of bio-organic fertilizer in disease prevention and control may be mainly attributed to its effect in the optimization of the physical and chemical properties and the microbial community of the rhizosphere soil [13,14,15,16].

It is well known that microbes are very important in soil ecosystems, and they can maintain soil fertility and decompose organic matter. The microbial community is the main factor affecting plant growth, and microbial communities are easily affected by fertilization. For example, organic fertilizer combined with chemical fertilizer significantly increases the utilization rate of carbon sources by soil microorganisms in double cropping paddy fields [17]. Furthermore, organic fertilizer application increased the organic matter and total nitrogen content of soil, as well as the relative contents of Rhodospiridae, Alphatrobacteria and Proteobacteria, and the yield of apple [8]. In addition, bio-organic fertilizer application not only activated different kinds of soil microbes, but also significantly improved the cucumber yield [15].

Soil metabolites also play an important role in the rhizosphere soil environment. Soil metabolites, including sugars, organic acids (such as amino acids and fatty acids) and secondary metabolites [18], are important substances that mediate the ecological relationship between plants and other organisms and can participate in plant growth, development, defense and other physiological processes. These substances play various roles in plant and microorganism interaction. Indeed, acid compounds show “low concentration promotion and high concentration inhibition” effects on plant seed germination and seedling growth and can also change the soil bacterial and fungal community structures [19,20]. Furthermore, some soil metabolites have toxic effects on plants, for example, esters can inhibit the respiration of plants, destroy the cell ultrastructure of plants, and reduce the richness of the bacterial community in rhizosphere soil [21].

Conversely, some soil metabolites are also beneficial to plants due to that they can change the chemical and physical properties of soil, control abiotic and biological processes, and regulate microbial communities [22]. For example, phenolic compounds are important secondary metabolites that play an important role in obtaining nutrients, allelopathy, regulating pH and enzyme activity and other ecological processes [23]. They not only participate in the scavenging process of plant reactive oxygen species, but also inhibit weeds and can be used to develop herbicides [24]. Umbelliferone can be used to develop new fungicides for natural products [25]. Rosmarinic acid can promote the attachment of beneficial bacteria to diatoms, while azelaic acid can promote the growth of beneficial bacteria and inhibit the growth of harmful bacteria [26]. The upregulation of polysaccharides, fatty acids and small dicarboxylic acids in rhizosphere soil treated with SiO_2_-NP might enrich the soil microbial community [22]. The upregulation of phenol and flavonoid metabolites can regulate nitrogen fixation and promote the growth of beneficial bacteria in maize rhizosphere soil [27]. Therefore, more attention should be paid on the role of root metabolites in plant growth and health enhancement.

We hypothesize that the decline disease can be alleviated by rhizosphere soil fertilization. In order to promote the sustainable development of the bayberry industry, the aim of this paper was to investigate the effects of compound fertilizer and bio-organic fertilizer on vegetative growth and fruit quality, as well as rhizosphere soil properties, microbial community structure and their metabolites. In addition, we examined the correlation of the major metabolites with microorganisms in rhizosphere soil of disease trees in presence or absence of the two fertilizer. This study provides a scientific basis to develop an effective measure for this management of bayberry decline disease by improving the microenvironment of rhizosphere soil.

## 2. Materials and Methods

### 2.1. Experimental Design

This experiment was carried out on fifteen-year-old trees of bayberry (cv. Dongkui) from the orchard located in Qianjiang Village (30°32′ N; 120°42′ E) of Huangwan Town, Haining City, Zhejiang Province, which was a typical gentle slope mountain area, approximately 50 m above sea level. The soil was acidic yellow in the orchard with the row spacing of 4 m × 5 m, while 65% of the bayberry plants were infected by decline disease with varied grades (1–9). The field was managed conventionally. The bayberry trees tested in this study exhibited similar loads, crown sizes, and disease index of grade 5 [4]. One kilogram of compound fertilizer (CF) (NPK 15-15-15) (Norwegian Hydro Co., Ltd.) was applied to each diseased tree at the flower bud differentiation period, and 20 kg of bio-organic fertilizer (sheep manure, N + P_2_O_5_ + K_2_O ≥ 5%, organic matter ≥ 45%, humid acid ≥ 7%, effective viable bacteria ≥ 0.2 billion·g^−1^) was applied to each tree at both the flower bud differentiation period and leaf flushing period. Deep ditches of 10–20 cm were dug continuously at the drip line of the tree crown (approximately 1.5 m from the tree trunk), and then the fertilizer was covered with fine soil. Three treatments were established in this study: declined disease alone (D), in combination with either compound fertilizer (CF) or bio-organic fertilizer (OF). The straight-line distance between each treatment was at least 100 m, and each treatment had 6 replicates, each replicate had 1 tree.

### 2.2. Measurement of Vegetative Growth Parameters

After about 8 months of fertilizer application, twenty branches of each treatment were randomly selected during the fruit mature period, the diameter of branches was measured using a digital Vernier caliper (Shanghai Daoju). The fourth to eighth leaves below the top of the vegetative branches in the middle part of the tree periphery were measured and sampled. Thirty leaves were selected for each index. The photosynthetic rates were measured by a LI-6400 portable photosynthesis instrument (LI-COR Company of America). The leaf length (from top to base of petiole) and the leaf width (the most) were measured using a ruler. The thickness was measured by a digital Vernier caliper, and the average value was calculated by repeating 6 times.

### 2.3. Measurement of Fruit Economic Characters

Two hundred mature fruits from each treatment were randomly selected during the fruit mature period. The weight, and soluble solids of a single fruit were determined immediately, and the samples were stored at −20 °C for the determination of the titratable acid and vitamin C contents of fruits. Fifteen fruits were weighed from each treatment with an electronic balance (Shanghai Precision Instrument), and the average value was taken. The contents of soluble solids (TSS) were determined by an ATAGOPR-101a hand-held digital glucometer (Japan). Titratable acid was determined by acid-base titration [28], while vitamin C was determined by 2–6 dichloroindophenol titration [29].

### 2.4. Soil Sample Collection and Physical and Chemical Property Measurements

The rhizosphere soil samples of 6 trees in each treatment were collected and repeated 6 times within one week after harvest of the fruits with stable vegetative and reproductive growth. Approximately 2 kg of mixed soil samples (0–20 cm) were collected at the drip line around the crown of the bayberry plant by using the quartering method and then passed through a 0.45-mm sieve. One sample was kept in a refrigerator at −80 °C for DNA extraction, while the other sample was dried at room temperature for soil properties measurement. After natural air-drying, soil physical and chemical properties were determined as follows. The pH was examined by using pH meter with a soil/water ratio of 1:2.5; organic matter was analyzed by the K_2_Cr_2_O_7_ oxidation method [30]; available N was measured using the alkaline hydrolysis diffusion method; available P was examined by the method of anti-molybdenum antimony colorimetry [31]; and exchangeable calcium and exchangeable magnesium were determined by using an ice3500 atomic absorption spectrophotometer following the extraction by ammonium acetate and iron [32].

### 2.5. Soil Genome Sequencing

Shanghai Ouyi Biomedical Technology Co., Ltd. was entrusted with the execution of genome sequencing. Genomic DNA of soil samples was extracted by a DNA extraction kit. The diluted genomic DNA was used as template. According to the selection of the sequencing region, specific primers with barcodes and Takara Ex Taq high-fidelity enzyme from the Takara company were used for PCR to ensure amplification efficiency and accuracy. Primers 343F-5′-TACGGAGGCAGAG-3′ and 798R-5′-AGGGTATCTATCT-3′ were used to amplify the 16S rRNA V3-V4 region of bacterial diversity [33]. Primers ITS1F-5′-CTTGGTCATTTAGGAAGTAA-3′ and ITS2-5′-GCTGCGTTCTTCATTCGATGC-3′ were used to amplify the corresponding regions ITS1 and ITS2 to determine fungal ITS diversity. The PCR products were sequenced using the Illumina MiSeqPE300 platform [34].

Paired-end reads were preprocessed as described in our recent publication [3] using Trimmomatic software [35] to cut off ambiguous bases and low-quality sequences and then assembled using FLASH software [36]. Further denoising was obtained by removing the reads with chimera, ambiguous, homologous sequences or less than 200 bp in length. Following primer sequences removal, clean reads were subjected to hierarchical clustering so as to generate operational taxonomic units (OTUs) using VSEARCH software with 97% similarity cutoff [37]. After the selection of the representative read of each OTU using QIIME package [38], all representative reads of both 16S rDNA and ITS were annotated and blasted against the Silva database (Version 123) using an RDP classifier (confidence threshold of 70%) [39] and the Unite database (ITSs rDNA) using BLAST [40], respectively. The alpha diversity was calculated using the Chao1 index [41] and Shannon index [42], while principal coordinates analysis (PCoA) and phylogenetic tree construction were carried out using the unweighted unifrac distance matrix obtained by QIIME.

### 2.6. Gas Chromatography-Mass Spectrometry (GC-MS) Metabolomics Analysis

The soil sample for GC-MS metabolomics analysis was prepared as described in our recent publication [3]. The reliability of the entire analysis was justified by inserting one quality control (QC) sample among every 10 samples. The QC sample was prepared by mixing the extract of all samples in equal volume, and the volume of each QC sample was the same as that of the tested sample. Furthermore, all soil samples were stored at room temperature for GC-MS metabolomics analysis, which was carried out and analyzed as described in our recent publication on a 7890B-5977A GC/MSD GC-MS (Agilent Technologies Inc., Santa Clara, CA, USA) [3]. The obtained data in this study were analyzed by comparing them with that of the standard spectrum library of the National Institute of Standards and Technology, while the obtained metabolite information in this study was searched against the KEGG database.

### 2.7. Statistical Analysis

R 3.5.1 was used for the principal coordinates analysis (PCoA), community histograms and redundancy discriminant analysis (RDA). OPLS-DA was made by R 3.6.2 ropls. The heat map was generated by using Pheatmap software. Preliminary data processing was performed by using Excel 2010, while the significance test (*p* < 0.05) and α-diversity metrics was calculated using SPSS 17.0 software (IBM, Chicago, IL, USA) and Kruskal–Wallis test, respectively.

## 3. Results and Discussion

### 3.1. The Effects of Compound/Bio-Organic Fertilizer on Vegetative Growth and Fruit Quality

The result from this study indicated that compared with the disease control, CF and OF treatments significantly affected all of the vegetative growth parameters except leaf length. Indeed, application of CF caused a 32.98%, 21.79%, 3.42%, 14.81% and 87.10% increase, respectively, while OF resulted in a 135.70%, 28.21%, 13.32%, 19.75% and 178.49% increase, respectively, in the twig length, twig diameter, leaf width, leaf thickness and photosynthetic rate compared to the disease control. Furthermore, OF exhibited a greater effect in all these parameters except leaf thickness, in which there was no significant difference between CF and OF (Table 1). This indicated that OF generally had a greater effect on vegetative growth compared to CF.

All fruit quality parameters of the disease control were significantly affected by both CF and OF. Indeed, CF resulted in a 28.29%, 12.35% and 38.90% increase in single fruit weight, soluble solids, and vitamin C, respectively, while OF caused a 57.03%, 38.50% and 44.96% increase in single fruit weight, soluble solids, and vitamin C, respectively, compared with the diseased control (Table 2). In contrast, the titratable acid contents of fruits from diseased trees were significantly reduced by CF (28.68%) and OF (44.96%), respectively (Table 2). In general, the result of this study revealed that OF exhibited a greater effect on improving fruit quality compared to CF. In agreement with the result of this study, OF is reported as able to not only promote the growth and yield of sweet potato, tea and tomato, but also to improve fruit quality [43,44,45].

### 3.2. The Effect of Compound/Bio-Organic Fertilizer in Microbial Community Diversity

**Figure 1:**
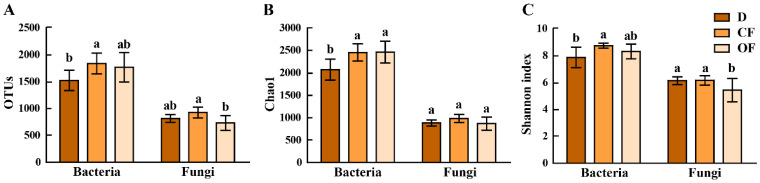
Effect of compound fertilizer and bio-organic fertilizer on OTU distribution (**A**) and the Chao1 indexes (**B**) and the Shannon’s diversity index (**C**) of bacteria and fungi in bayberry rhizosphere soil. D, CF and OF represents decline disease alone, in combination with either compound fertilizer or bio-organic fertilizer, respectively. Values with different lowercase letters within the same treatments indicate significant differences (*p* < 0.05).

The two fertilizers caused a significant increase in the number of operational taxonomic units (OTUs) and Chao1 index in the bacterial V3 + V4 region compared with the disease control. The average bacterial OTUs number of diseased trees alone, in combination with either CF or OF is 1575.33 (vary from 1203 to 1781), 1833.33 (vary from 1518 to 2078) and 1765.67 (vary from 1454 to 2114), respectively. In general, CF and OF caused 16.38% and 12.08%, respectively, increase in the bacterial OTUs number, 18.52% and 18.87%, respectively, increase in bacterial Chao1 index, 10.94% and 5.60%, respectively, increase in bacterial Shannon index of rhizosphere soil microbial community compared to that of diseased trees alone (Figure 1).

The average fungal OTUs number of diseased trees alone, in combination with either CF or OF is 814.17 (varying from 725 to 884), 925.33 (varying from 818 to 1069) and 730.50 (varying from 568 to 933), respectively. In general, CF caused 13.65% and 11.45%, respectively, in the average fungal OTUs number and Chao1 indexes, while OF caused 10.30% and 11.44%, respectively, reduction in the average fungal OTUs number and Shannon index compared to that of the diseased control. The fungal Chao1 of and Shannon indexes were unaffected by CF. Furthermore, the average bacterial OTUs number is 1.93-, 1.98- and 2.42-fold greater than that of fungi in diseased control, CF and OF treatments, respectively. The bacterial Chao1 indexes is 2.34-, 2.49-, and 2.84-fold greater than that of fungi in diseased control, CF and OF treatments, respectively. Similarly, bacterial Shannon indexes is 1.28-, 1.42-, and 1.53-fold greater than that of fungi in diseased control, CF and OF treatments, respectively. In addition, compared to CF treatment, OF treatment caused a greater ratio of bacterial and fungal OTU distribution and diversity indexes. The results indicated that diversity of bacterial in rhizosphere soil was significantly increased, and the diversity of fungi was significantly reduced by OF treatment (Figure 1).

In agreement with the results of this study, the role of fertilizer, in particular bio-organic fertilizer, in soil microbe and plant growth is reported in many previous studies [33,45,46,47]. For example, the long-term application of organic fertilizer increased the species of bacteria in kiwifruit rhizosphere soil [46]. The total bacterial richness (the number of species in the community) was increased by 70% after application of bio-organic fertilizer in combination with 30% chemical fertilizer [45]. Furthermore, it is reported that the bacterial Chao1 index and Shannon index of rhizosphere soil was able to be significantly improved by bio-organic fertilizer [33]. The rhizosphere microbiome was reshaped by bio-organic amendment, resulting in the increase in crop yield [47].

### 3.3. The Effect of Compound/Bio-Organic Fertilizer in Soil Microbial Community Structure

**Figure 2:**
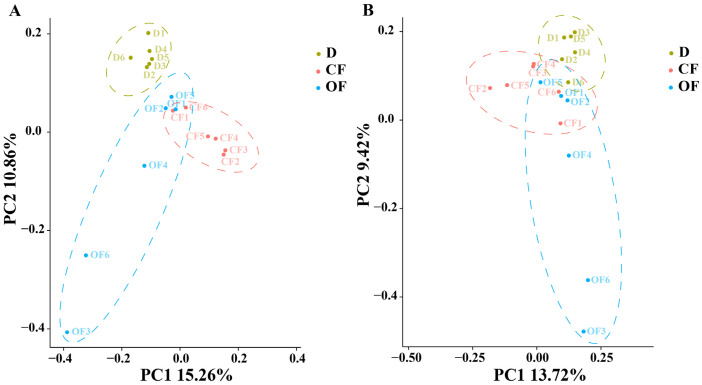
PCoA results of soil bacteria (**A**) and fungi (**B**) based on OTU abundance. D, CF, and OF represented decline diseased trees, in combination with either compound fertilizer or bio-organic fertilizer. Effect of compound/bio-organic fertilizer on soil microbial community composition.

PCoA analysis of bacterial community structure indicated that the six replicates of the disease control, CF and OF were divided into three different groups, while the disease control was well separated from the treatments of CF or OF, indicating that the microbial community structure of rhizosphere soil was significantly changed by both CF and OF (Figure 2A). Similarly, PCoA analysis of fungal community structure indicated that six replicates of the disease control, CF and OF were divided into three different groups, however, there was an overlap among the disease control, CF and OF (Figure 2B). In general, a greater diversity was also observed in six replicates of the OF treatment compared to that of the disease control and the CF treatment regardless of the bacterial or fungal community structure (Figure 2). In agreement with the results of this research, after three years of continuous application of the OF treatment caused a significant change in the principal coordinates of bacterial and fungal communities in cotton *Verticillium* wilt soil [48].

**Figure 3:**
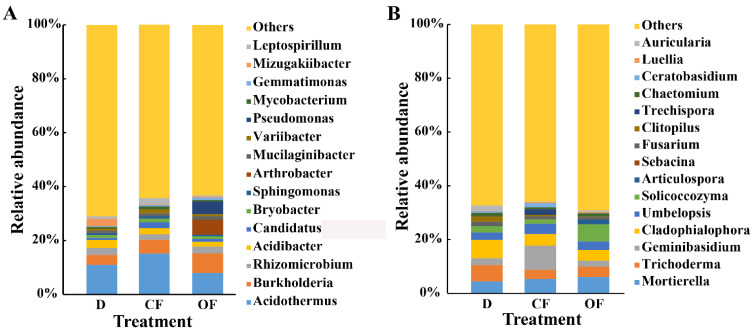
Relative abundance of bacteria (**A**) and fungi (**B**) at the genus level. D, CF, and OF represented decline diseased trees, in combination with either compound fertilizer or bio-organic fertilizer, respectively.

This result indicated that the application of CF and OF resulted in a significant change in the composition of the bacterial and fungal community at the phylum (Figure S1), order (Figure S2) and genus levels compared to the diseased control (Figure 3). Indeed, the top 15 species in the rhizosphere soil of bayberry were selected to generate a relative abundance histogram, in which *Acidothermus*, *Burkholderia*, *Rhizomicrobium* and *Acidibacter* were the main bacterial genera (average relative abundance > 1%). Compared with that of the diseased control, the CF and OF treatments caused 38.97% and 99.48% increase in the relative abundance of *Burkholderia*, 23.48% and 8.03% increase in the relative abundance of *Rhizomicrobium*, and a 22.81% and 37.30% reduction in *Acidibacter*, respectively, in rhizosphere soil (Figure 3A). In particular, more attentions should be paid on *Burkholderia*, which are reported to play a role in plant growth by fixing nitrogen and enhancing the antioxidant defense system, the activities of soil urease, phosphatase, sucrase and catalase, as well as the expression of tillering response genes, thus reducing the harmful abiotic stress effect [49,50].

According to the distribution and relative abundances of fungi in bayberry rhizosphere soil at the genus level, *Mortierella*, *Trichoderma*, *Geminibasidium* and *Cladophialophora* were the main genera (average relative abundance > 1%), and they were also among the top 15 dominant fungal genera in terms of relative abundance, accounting for more than 75% of the fungal sequences, with high relative abundance (Figure 3B). Compared with the diseased control, the CF and OF treatments caused a 20.22% and 36.80% increase in the relative abundance of *Mortierella* in rhizosphere soil, a 44.34% and 37.49% reduction in *Trichoderma*, and a 35.95% and 39.46% reduction in *Cladophialophora*. Interestingly, the relative abundance of *Geminibasidium* were increased by 251.79% in CF treatment, but reduced by 13.99% in OF treatment. There was no report about the role of *Geminibasidium* and *Cladophialophora* in plant growth. However, *Mortierella* was reported to be beneficial to plant growth, for example, *Mortierella* in the rhizosphere of wheat is significantly associated with increasing crop growth and yield [51], while *Trichoderma* can be considered as an important biocontrol fungus by protecting crop against pathogen infection [52]. The increase in relative abundance of *Mortierella* may promote plant growth.

### 3.4. The Effect of Compound/Bio-Organic Fertilizer on the Soil Nutrient Status

CF and OF treatments exhibited a differential effect on the pH, physical and chemical properties of bayberry rhizosphere soil. Indeed, compared with the disease control, the content of organic matter was unaffected by CF, but was significantly increased by OF (26.40%). The content of alkali-hydrolyzable nitrogen was significantly reduced by CF (12.24%), but unaffected by OF; the available phosphorus was unaffected by CF, but was significantly reduced by OF (47.87%), the exchangeable calcium was significantly reduced by CF (9.38%), but was significantly increased by OF (142.31%), and the available Mg was significantly reduced by CF (9.63%), but was significantly increased by OF (12.73%) (Table 3). In addition, the pH of soils in the disease control was unchanged by both CF and OF treatments.

The results indicated that the pH, organic matter content and available phosphorus was unaffected by CF, which caused a negative effect in exchangeable magnesium, alkali-hydrolyzable nitrogen and exchangeable calcium. In contrast, the pH, alkali-hydrolyzable nitrogen was unaffected by OF, which caused an increase in organic matter content, exchangeable magnesium, and exchangeable calcium, but a significant reduction in available phosphorus. In agreement with the result of this study, previous studies revealed that the contents of available phosphorus in soil from the diseased trees were significantly increased compared to the healthy trees. Furthermore, it is well known that the organic matter have been proposed to exhibit greater effect on microbial communities compared to the other soil parameters [28,29,30,31,32]. Therefore, the greater effect of OF than CF in rejuvenation of diseased bayberry trees may be able to be, at least partially, attributed to the increase in organic matter.

### 3.5. The Effect of Compound/Bio-Organic Fertilizer on RDA of Soil Properties and Microbial Communities

**Figure 4:**
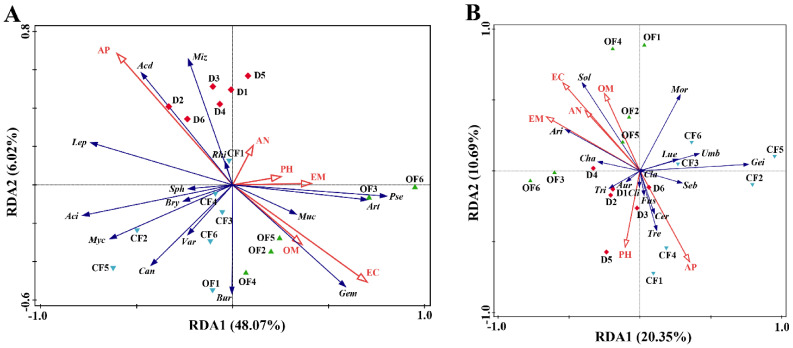
RDA of the rhizosphere microbial community composition at genus levels with soil physiological and chemical properties. (**A**): Baceria. *Acd*, *Acidibacter*; *Aci*, *Acidothermusl*; *Art*, *Arthrobacter*; *Bry*, *Bryobacter*; *Bur*, *Burkholderia*; *Can*, *Candidatus_solibacter*; *Muc*, *Mucilaginibacter*; *Myc*, *Mycobacterium*; *Pse*, *Pseudomonas*; *Rhi*, *Rhizomicrobium*; *Sph*, *Sphingomonas*; *Var*, *Variibacter*. (**B**) Fungi. *Gem*, *Gemmatimonas*; *Ari*, *Articulospora*; *Aur*, *Auricularia*; *Cer*, *Ceratobasidium*; *Cha*, *Chaetomium*; *Cla*, *Cladophialophora*; *Cli*, *Clitopilus*; *Fus*, *Fusarium*; *Gmi*, *Geminibasidium*; *Lep*, *Leptospirillum*; *Lue*, *Luellia*; *Miz*, *Mizugakiibacter*; *Mor*, *Mortierella*; *Seb*, *Sebacina*; *Sol*, *Solicoccozyma*; *Tre*, *Trechispora*; *Tri*, *Trichoderma*; *Umb*, *Umbelopsis*. RDA: Redundancy discriminant analysis. PH: pH; OM: organic matter; AN: alkali-hydrolyzable nitrogen; AP: available phosphorus; EC: exchangeable calcium; EM: exchangeable magnesium. Red diamond: samples from Decline bayberry; Blue down triangle: sample from compound fertilizer treated trees (CF); Green up triangle: sample from bio-organic fertilizer treated trees (OF).

Soil properties exhibited an influence in the composition of bacterial and fungal communities in bayberry rhizosphere soil at the phylum (Figure S3; Table S1) and genus levels (Figure 4; Table 4). Results from this study showed that there was a total of 54.09% and 31.04% of the cumulative variance of the rhizosphere microbial community-factor correction at the bacterial (Figure 4A) and fungal (Figure 4B) genus level, respectively. The contributions of the two main variables, exchangeable calcium and available phosphorus, explained 25.8% and 20.6% of the bacterial community at the genus level, respectively (Figure 4A), while the contributions of the two main variables, exchangeable magnesium and calcium, explained 12.3% and 11.0% of the fungal community at the genus level, respectively (Figure 4B).

Furthermore, the result of this study showed that there was a complex relationship between soil parameters and plant/microbial growth and soil nutrient elements because there was a differential change between the disease alone and in combination with either CF or OF in the contents of available soil nutrient elements, such as the content of alkali-hydrolyzable nitrogen, exchangeable calcium, available phosphorus and exchangeable magnesium in bayberry rhizosphere soil. In agreement with the result of this study, the growth of soil microbe is affected by a lot of environmental factors such as soil pH and organic matter as well as the content of exchangeable magnesium, nitrogen, available phosphorus and exchangeable calcium [53].

### 3.6. The Effect of Compound/Bio-Organic Fertilizer on Rhizosphere Soil Metabolomics

**Figure 5:**
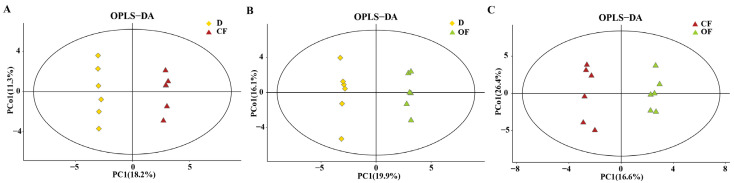
OPLS-DA score map of bayberry rhizosphere soil of the compound fertilizer treatment and bio-organic fertilizer treatment. (**A**) D and CF, (**B**) D and OF. (**C**) CF and OF. D, CF and OF represents decline disease alone, in combination with either compound fertilizer, or bio-organic fertilizer. Effect of compound/bio-organic fertilizer on differential metabolites analysis.

Following the identification of a total of 223 metabonomics based on GC-MS analysis of bayberry rhizosphere soils with different treatments, a score map of metabolites was achieved (Figure 5) by using the orthogonal partial least squares discriminant analysis (OPLS-DA), which is regarded as a supervised statistical method of discriminant analysis [3]. This result indicates that metabonomics in the disease control could be effectively separately between the disease control and CF or OF. Indeed, Figure 5A presents the distribution of the sample points of the disease control and the CF treatment in the negative and positive area of t[1], respectively, while the model values of D and CF were R^2^X(cum) = 0.395, R^2^Y(cum) = 0.9999, Q^2^(cum) = 0.803, R^2^ values were 0.982, Q^2^ values were −0.156. similarly, Figure 5B presents the distribution of the sample points of the disease control and the OF treatment in the negative and positive, respectively, value area of t[1], while the model values of D and OF were R^2^X(cum) = 0.461, R^2^Y (cum) = 0.998, Q^2^ (cum) = 0.805, R^2^ values were 0.845, and Q^2^ values were −0.185. Furthermore, the metabonomics in the CF treatment could be effectively separately from that of the OF treatment. Indeed, Figure 5C presents the distribution of the sample points of CF and OF in the negative and positive value area of t[1], respectively, while the model values of CF and OF were R^2^X(cum) = 0.549, R^2^Y (cum) = 0.988, Q^2^ (cum) = 0.686, R^2^ values were 0.827, Q^2^ values were −0.253. The results showed that the interpretation and prediction ability of the three groups of models were good due to that the R^2^ values were greater than 0.5. Therefore, it can be inferred from the obvious separation of samples that the types of metabolites in diseased rhizosphere soil were significantly changed by the application of the CF and OF treatment, which were also different from each other.

**Figure 6:**
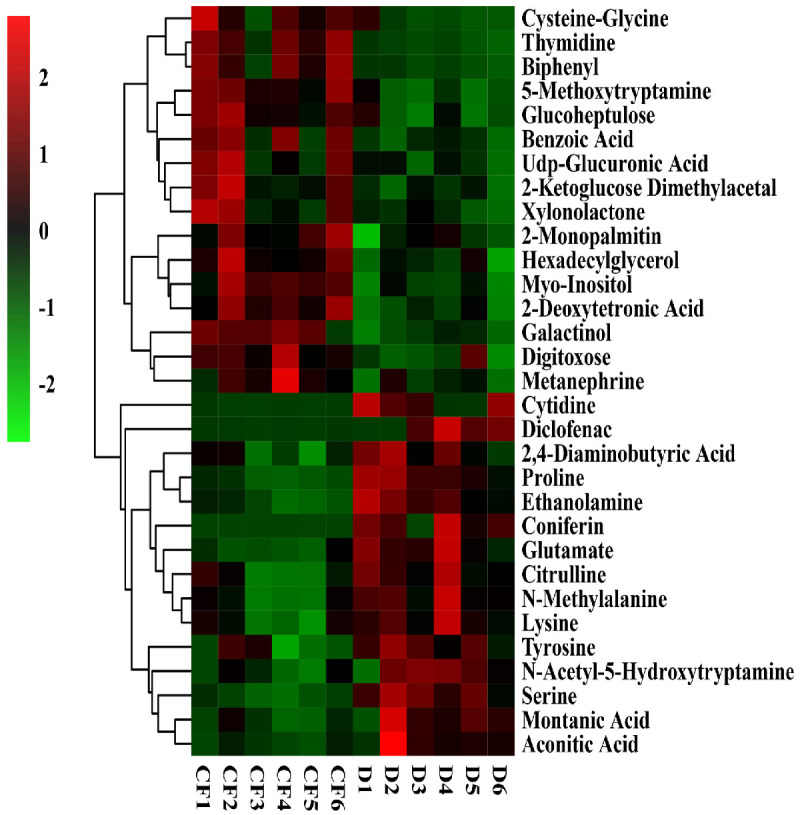
Thermogram analysis of different metabolites in bayberry rhizosphere soil of the compound fertilizer treatment. D and CF represented decline diseased trees alone or in combination with compound fertilizer, respectively.

**Figure 7:**
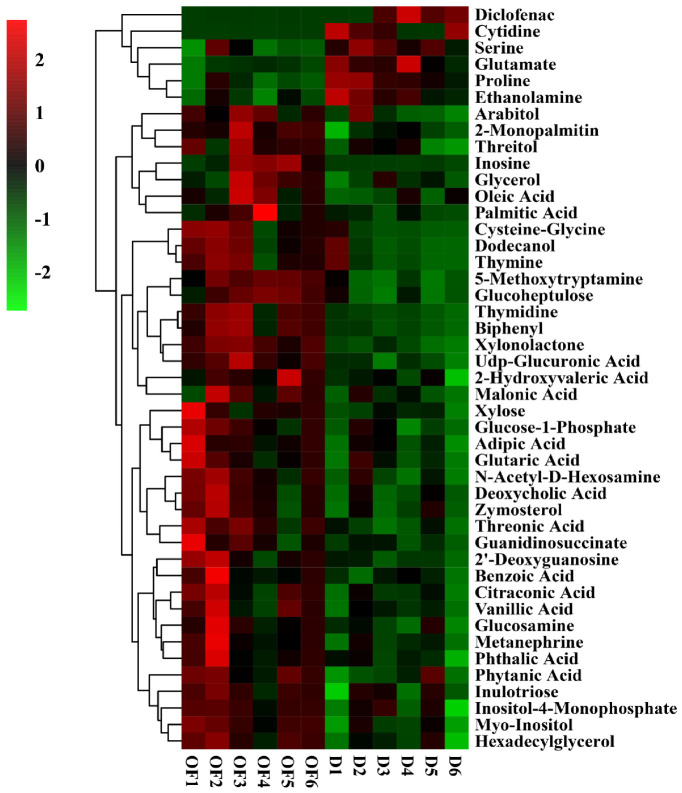
Thermogram analysis of different metabolites in bayberry rhizosphere soil of the bio-organic fertilizer treatment. D and OF represented decline diseased trees alone or in combination with bio-organic fertilizer, respectively.

Result from this study indicated that there was a difference in the relative contents of metabolites between the disease control and the CF or OF treatments (Figure 6 and Figure 7). Compared with the control, 31 metabolites was significantly changed by CF, with the increase of 31.13–250.00% in 16 metabolites and the decrease of 28.72–97.10% in the contents of 15 metabolites (Figure 6). Compared with the control, 45 different metabolites were significantly changed by OF with the increase of 23.81–390.77% in 39 metabolites and the decrease of 52.09–95.87% in the contents of six metabolites (Figure 7).

Compared to the control, both CF and OF significantly increased the relative levels of 12 metabolites including cysteine-glycine, thymidine, biphenyl, 5-methoxytryptamine, glucoheptulose, benzoic acid and UDP-glucuronic acid, xylonolactone, 2-monopalmitin, hexadecylglycerol, myo-inositol and metanephrine (Table 5). In particular, the content of inositol was increased by CF (72.96%) and OF (47.31%), respectively. Interestingly, inositol that is a biological mimic of guanosine, can participate in material metabolism and energy metabolism, exhibit good cell membrane permeability, and improve the activities of various enzymes [54]. In contrast, CF and OF significantly reduced the relative contents of six metabolites, diclofenac, cytidine, serine, glutamate, proline and ethanolamine compared to the disease control (Table 5). In particular, the content of diclofenac was decreased by CF (95.74%) and OF (95.57%), respectively. Diclofenac seriously affects the growth and development of plants by inhibiting the activity of the mitochondrial respiratory electron transport chain and the pathway of respiratory carbon metabolism, and causing abiotic stress and disorders of material metabolism and energy metabolism in cells, which will lead to an increase in the activity of glutathione-S-transferase in roots [55].

Obviously, the CF and OF treatments exhibited a differential effect in the change of substances compared to the disease control (Figure 6 and Figure 7). Indeed, 13 metabolites were changed by CF but not OF, among them, 2-ketoglucose imethylacetal, 2-deoxytetronic acid, galactinol and digitoxose were increased, while 9 substances such as 2, 4-diaminobutyric acid, coniferin, citrulline, N-methylalanine, lysine, tyrosine, montanic acid, aconitic acid and N-acetyl-5-hydroxytryptamine were reduced (Table 6). In contrast, 27 substances were increased by OF but not CF, including arabitol, threitol, inosine, glycerol, oleic acid, palmitic acid, dodecanol, thymine, 2-hydroxyvaleric acid, xylose, glucose-1-phosphate, glutaric acid, N-acetyl-D-hexosamine, deoxycholic acid, zymosterol, threonic acid, guanidinosuccinate, 2’ -deoxyguanosine, citraconic acid, vanillic acid, glucosamine, phthalic acid, phytanic acid, inulotriose, inositol-4-monophosphate, malonic acid, and adipic acid were significantly increased (Table 7). In agreement with the result of this study, the application of bio-organic fertilizer caused the increase in the relative content of glucoheptulose, palmitic acid, phytanic acid, and deoxycholic acid [56,57].

Among the metabolites, inosine is reported to be essential for the growth of plants, which is involved in phosphatidylinositol signal transduction, auxin storage and transportation, phytic acid biosynthesis, cell wall biosynthesis and plant response to stress [58], and it can protect the cell membrane system by reducing the damage of membrane lipid peroxidation and enhancing the activity of antioxidant enzymes [34,54]. Furthermore, amino acids are not only a substrate for protein synthesis but can also induce plant growth, promote the recovery of a normal metabolism and osmotic pressure balance of plants from stress, and promote plant growth through rhizosphere bacteria [22,59]. In general, the result revealed that the metabolites in the rhizosphere soil were differentially affected by OF and CF, while the former exhibited a greater effect than that of the latter. Thus, it can be inferred that the different effects of CF and OF on plants and rhizosphere microorganisms may be reflected through the influence of different substances.

### 3.7. The Effect of Compound/Bio-Organic Fertilizer on the Metabolic Pathways

**Figure 8:**
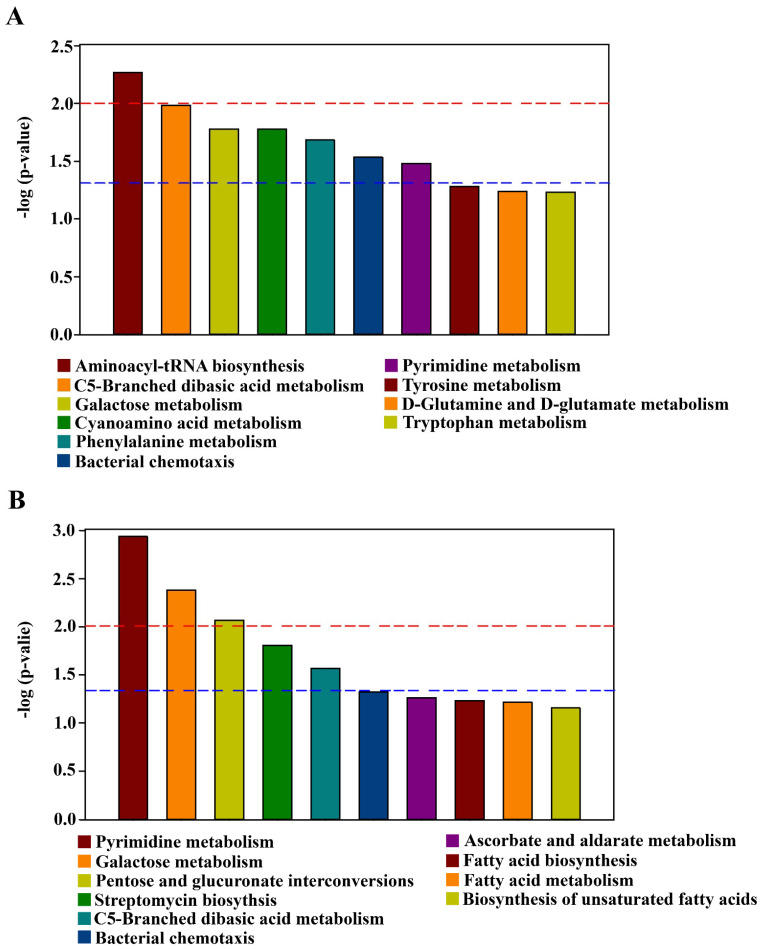
Metabolic pathway enrichment map of different metabolites in bayberry rhizosphere soil of the compound fertilizer treatment and bio-organic fertilizer treatment. (**A**) D and CF, (**B**) D and OF. D, CF, and OF represented decline diseased trees, in combination with either compound fertilizer or bio-organic fertilizer, respectively. The signal pathway is significant when the top of the bar is higher than the blue (*p* < 0.05) or red line (*p* < 0.01).

The pathway enrichment analysis of different metabolites were conducted with the KEGG (Kyoto Encyclopedia of Genes and Genomes) database. Among the ten obtained pathways, CF caused significant change in seven metabolic pathways, including aminoacyl-tRNA biosynthesis, C5-branched dibasic acid metabolism, galactose metabolism, cyanoamino acid metabolism, phenylalanine metabolism, bacterial chemotaxis, and pyrimidine metabolism (Figure 8). The aminoacyl-tRNA biosynthesis is one essential for protein biosynthesis, and has significant impact on growth performance in *Macrobrachium rosenbergii* [60]. In higher plants, the amino acid phenylalanine is a substrate of both primary and secondary metabolic pathways. The primary pathway that consumes phenylalanine, protein biosynthesis, is essential for the viability of all cells [61]. Both primary and secondary metabolic pathways are considered to be important in ecologically relevant conditions, where chemotaxis is normally coupled with metabolism and growth, due to the presence of diverse chemoattractant cues and their active consumption by various types of bacteria [62].

Among the ten obtained pathways, OF caused significant change in five metabolic pathways, including pyrimidine metabolism, galactose metabolism, pentose and glucuronate interconversions, streptomycin biosynthesis, and C_5_-branched dibasic acid metabolism (Figure 8). Interestingly, the pentose and glucuronate interconversions pathway was shared by both *Botrytis cinerea* and *Bradysia odoriphaga* [63]. Furthermore, Streptomycin is one kind of antibiotics, which could be acted as both an offensive and defensive weapon that facilitates invasion into occupied habitats and also protects against invasion by competitors [64]. Therefore, it can be inferred that fertilization may have an influence on plant pathogen and insects of rhizosphere soil.

The three pathways including aminoacyl-tRNA biosynthesis, cyanoamino acid metabolism, and phenylalanine metabolism were changed by CF, but not OF (Figure 8A); while the two pathways including pentose and glucuronate interconversions, and streptomycin biosynthesis were changed by OF, but not in CF (Figure 8B). Notably, three pathways, including C5-branched dibasic acid metabolism, galactose metabolism, and pyrimidine metabolism, were significantly changed by both CF and OF compared with the control (Figure 8). Interestingly, galactose is crucial for human metabolism, with an established role in energy delivery and galactosylation of complex molecules, and evidence for other roles is emerging [65]. Pyrimidine pathway is a key metabolic pathway, resulting in the formation of pyrimidines, then integration into nucleic acids, sugars and lipids [66]. This indicated that these metabolic pathways may be sensitive to the two fertilizers.

### 3.8. The Correlation of Soil Microorganisms with Metabolites

**Figure 9:**
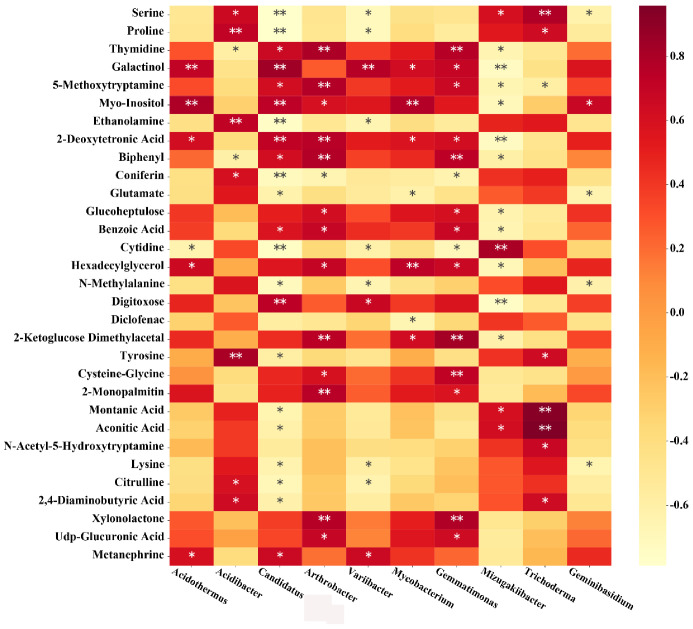
Correlation analysis between the microorganism relative abundances at the genus level and the metabolite relative contents of the compound fertilizer treatment. * and ** represents a significant correlation at *p* < 0.05 and *p* < 0.01, respectively. The magnitude of the correlation coefficient was indicated by the depth of the orange scale, while the darker color is the greater positive correlation, and the lighter color is the greater negative correlation.

**Figure 10:**
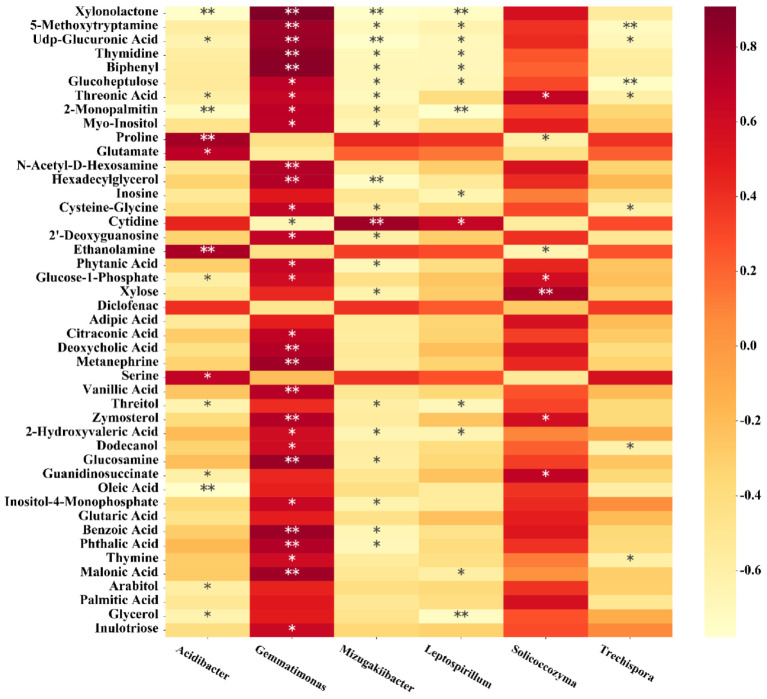
Correlation analysis between soil bacterial and fungal relative abundances at the genus level and the metabolite relative contents of the bio-organic fertilizer treatment. * and ** represents a significant (*p* < 0.05 and *p* < 0.01, respectively) correlation. The magnitude of the correlation coefficient was indicated by the depth of the orange scale, while the darker color is the greater positive correlation, and the lighter color is the greater negative correlation.

This result revealed a correlation between microbial groups at the phylum, order and genus levels and secondary metabolites of bayberry rhizosphere soil regardless of the presence or absence of the two fertilizers (Figure 9 and Figure 10, Supplementary Figures S4–S7). In general, the increase in main metabolites (sugars and organic acids) and secondary metabolites by the application of CF or OF in the rhizosphere soil of decline diseased trees may enrich the soil microbiota and help coordinate the rhizosphere microbiota. The decrease in proline, cytidine and diclofenac may be the result of a reduction in the number of harmful microorganisms in the rhizosphere soil of declined trees. Therefore, the effect of fertilization on soil microorganism may be, at least partially, attributed to the change of metabolites in rhizosphere soil of declined disease bayberry.

In CF treatment, at the genus level, *Acidothermus* was significantly positively correlated with galactinol, myo-inositol, 2-deoxytronic acid, hexadecyllycerol and metanephrine, and negatively correlated with cytidine. *Acidibacter* was significantly positively correlated with serine, proline, ethanolamine, coniferin, tyrosine, citrulline and 2,4-diaminobutyric acid, and significantly negatively correlated with thymidine and biphenyl. There was a significant positive correlation between *Candidatus solibacter* and thymidine, galactinol, 5-methoxytryptamine, myo-inositol biphenyl, 2-deoxytronic acid, biphenyl, benzoic acid, digitoxose and metanephrine, and significant negative correlated with serine, proline, ethanolamine, coniferin, glutamate, cytidine, N-methylalanine, tyrosine, montanic acid, acidic acid, lysine, citrulline and 2,4-diaminobutyric acid. *Arthrobacter* was positively correlated with thymidine, 5-methoxytryptamine, myo-inositol, 2-deoxytronic acid, biphenyl, glucoheptulose, benzoic acid, hexadecylglycerol, 2-ketoglucose, cystine glycine, 2-monopalmitin, xylonolactone and UDP gluconic acid, and negatively correlated with coniferin. *Variibacter* was positively correlated with galactinol, digitoxose and metanephrine, and negatively correlated with serine, proline, ethanolamine, cytidine, N-methylalanine, lysine and citrulline. *Mycobacterium* was significantly positively correlated with galactinol, myo-inositol, 2-deoxytronic acid, hexadecyllycerol and 2-ketoglucose dimethylacetal, and negatively correlated with glutamate and diclofenac. *Gemmatimonas* was significantly positively correlated with thymidine, galactinol, 5-methoxytryptamine, 2-deoxytronic acid, biphenyl, glucoheptulose, benzoic acid, hexadecylglycerol, 2-ketoglucose dimethylacetal, cysteine glycine, 2-monopalmitin, xylonolactone and UDP glucuronic acid, and significant negatively correlated with coniferin and cytidine. *Mizugakiibacter* was significantly positively correlated with serine and cytidine, montanic acid and acidic acid, and significantly negatively correlated with thymine, galactinol, 5-methoxytryptamine, myo-inositol, 2-deoxytronic acid, biphenyl, glucoheptulose, benzoic acid, hexadecylglycerol, digitoxose and 2-ketoglucose dimethylacetal. *Trichoderma* in fungi was significantly positively correlated with serine, proline, montanic acid, acidic acid, n-acetyl-5-hydroxytryptamine and 2,4-diaminobutyric acid, and significantly negatively correlated with 5-methoxytryptamine. *Geminibasidium* was significantly positively correlated with myo-inositol, and negatively correlated with serine, glutamate, N-methylalanine and lysine (Figure 9).

In the OF treatment, at the genus level, *Acidibacter* was significantly positively correlated with proline, glutamate, ethanolamine and serine, and negatively correlated with xylnolactone, UDP glucuronic acid, threonic acid, 2-monopalmitin, glucose-1-phosphate, threitol, guanidinosuccinate, oleic acid, arabitol and glycerol. *Gemmatimonas* were significantly positively correlated with xylolactone, 5-methoxytryptamine, UDP gluconic acid, thymidine, biphenyl, glucoheptulose, threonic acid, 2-monopalmitin, myo-inositol, n-acetyl-d-hexosamine, hexadecylglycerol, cysteine glycine, 2′- deoxyguanosine, phytic acid, glucose-1-phosphate, citric acid, deoxycholic acid, metanephrine vanillic acid, zymosterol, 2-hydroxyvaleric acid, dodecanol, glucosamine, inositol-4-monophosphate, benzoic acid, phthalic acid, thymine, malonic acid and inulotriose, and were negatively correlated with cytidine (Figure 10). *Mizugakiibacter* was significantly positively correlated with cytidine, and was significantly negatively correlated with xylolactone, 5-methoxytryptamine, UDP gluconic acid, thymidine, biphenyl, glucoheptulose, threonic acid, 2-monopalmitin, myo-inositol, hexadecylglycerol, cystine glycine, 2′-deoxyguanosine, phytic acid, xylose, threitol, 2′-hydroxyvaleric acid, glucosamine, inositol-4-monophosphate, benzoic acid and phthalic acid (Figure 10). *Leptospirillum* was positively correlated with cytidine, and negatively correlated with xylonolactone, 5-methoxytryptamine, UDP gluconic acid, thymidine, biphenyl, glucoheptulose, 2-monopalmitin, inosine, threitol, 2′-hydroxyvaleric acid, malonic acid and glycerol (Figure 10). *Solidocozyma* in fungi was significantly positively correlated with threonic acid, glucose-1-phosphate, xylose, zymosterol and guanidinosuccinate, and significantly negatively correlated with proline and ethanolamine (Figure 10). *Trechispora* was no significant positive correlation with the 45 listed metabolites, and significantly negatively correlated with 5-methoxytryptamine, UDP gluconic acid, glucoheptulose, threonic acid, cystine glycine, dodecanol, and thyminen (Figure 10). This shows that after the application of OF fertilizer, soil microorganisms are closely related to secondary metabolites at the phylum, order and genus levels.

## 4. Conclusions

The results of this study showed that OF had a greater effect on the rejuvenation of the decline disease trees, and had a better effect on improving soil organic matter content compared with CF. Furthermore, the composition of microbial communities in bayberry rhizosphere soil was affected significantly by exchangeable calcium, available phosphorus and exchangeable magnesium at the genus level, and the three main variables had a greater effect in bacterial communities than fungal communities. Compared with the disease control, CF and OF caused a significant increase in the relative abundance of *Burkholderia* and *Mortierella*, and a reduction in the relative abundance of *Rhizomicrobium* and *Acidibacter* and *Trichoderma* and *Cladophialophora*, while the relative abundance of *Geminibasidium* was increased by CF, but reduced by OF. In particular, OF resulted in a greater change in the kinds and the relative contents of metabolites in the rhizosphere soil compared to CF. In addition, CF and OF caused significant change of seven and five metabolic pathways, respectively, while both of them contain galactose metabolism and pyrimidine metabolism. The result revealed a significant correlation at the phylum, order and genus levels between microbial groups and secondary metabolites of bayberry rhizosphere soil regardless of the two fertilizers. In summary, the results revealed the influence of the two fertilizers in plant growth and fruit quality as well as rhizosphere soil properties, microbiota, and secondary metabolites, which provides a new insight to reduce the damage of this decline disease to bayberry.

## Figures and Tables

**Table 1:** Effects of compound/bio-organic fertilizer on vegetative growth of decline disease bayberry.

Parameters	Value	Parameters	Value
Twig length (cm)		Twig diameter (mm)	
D	13.22 ± 0.54 ^c^	D	2.80 ± 0.13 ^c^
CF	17.58 ± 0.55 ^b^	CF	3.41 ± 0.08 ^b^
OF	31.16 ± 0.40 ^a^	OF	3.59 ± 0.18 ^a^
Leaf length (mm)		Leaf width (mm)	
D	99.50 ± 3.85 ^a^	D	27.18 ± 0.41 ^c^
CF	99.11 ± 5.98 ^a^	CF	28.11 ± 0.34 ^b^
OF	97.31 ± 7.62 ^a^	OF	30.80 ± 0.63 ^a^
Leaf thickness (mm)		Photosynthesis rate (mg CO_2__·_∙10 cm^−2^∙h^−1^)	
D	4.05 ± 0.92 ^b^	D	1.86 ± 0.28 ^c^
CF	4.65 ± 0.18 ^a^	CF	3.48 ± 0.21 ^b^
OF	4.85 ± 0.30 ^a^	OF	5.18 ± 0.32 ^a^

D, CF and OF represents decline disease alone, in combination with either compound fertilizer or bio-organic fertilizer, respectively. Different lowercase letters within the same parameters indicate significant differences (*p* < 0.05).

**Table 2:** Effects of compound fertilizer and bio-organic fertilizer on fruit quality of decline disease bayberry.

Parameters	Value	Parameters	Value
Single fruit weight (g)		Soluble solids (%)	
D	11.03 ± 0.51 ^c^	D	8.91 ± 0.29 ^c^
CF	14.15 ± 0.30 ^b^	CF	10.01 ± 0.26 ^b^
OF	17.32 ± 1.06 ^a^	OF	12.34 ± 0.13 ^a^
Vitamin C (mg/100 g)		Titratable acid (%)	
D	4.73 ± 0.17 ^c^	D	1.29 ± 0.11 ^a^
CF	6.57 ± 0.58 ^b^	CF	0.92 ± 0.07 ^b^
OF	8.06 ± 2.04 ^a^	OF	0.71 ± 0.07 ^c^

D, CF and OF represents decline disease alone, in combination with either compound fertilizer or bio-organic fertilizer, respectively. Values with different lowercase letters within the same parameters indicates significant differences (*p* < 0.05).

**Table 3:** Effect of compound fertilizer and bio-organic fertilizer on rhizosphere soil pH, physical and chemical properties of decline disease bayberry.

Physical and Chemical Properties	D	CF	OF
pH	4.22 ± 0.01 ^a^	4.36 ± 0.37 ^a^	4.34 ± 0.21 ^a^
Organic matter (%)	2.50 ± 0.86 ^b^	2.37 ± 0.13 ^b^	3.16 ± 0.35 ^a^
Alkali-hydrolyzable nitrogen (mg/kg)	106.11 ± 3.30 ^a^	93.12 ± 4.27 ^b^	105.15 ± 3.40 ^a^
Available phosphorus (mg/kg)	27.72 ± 0.31 ^a^	25.31 ± 0.74 ^a^	14.45 ± 0.45 ^b^
Exchangeable calcium (mg/kg)	315.16 ± 3.95 ^b^	285.61 ± 2.37 ^c^	763.66 ± 18.08 ^a^
Exchangeable magnesium (mg/kg)	31.98 ± 0.35 ^b^	28.90 ± 2.80 ^c^	36.05 ± 2.57 ^a^

D, CF and OF represents decline disease alone, in combination with either compound fertilizer, or bio-organic fertilizer, respectively. Values with different lowercase letters within the same treatments indicates significant differences (*p* < 0.05).

**Table 4:** Contribution of soil properties to bacterial and fungal taxa at the genus level.

Soil Environment	Contribution at Bacterial Genus Level (%)	Contribution at Fungal Genus Level (%)
pH	3.9	6.0
Organic matter	7.3	6.6
Alkali-hydrolyzable nitrogen	3.2	8.5
Available phosphorus	20.6	8.6
Exchangeable calcium	25.8	11.0
Exchangeable magnesium	10.2	12.3

**Table 5:** The relative contents of metabolites in rhizosphere soil of diseased bayberry trees was increased by both compound fertilizer and bio-organic fertilizer.

No.	Metabolite Name	D	CF	OF
1	Cysteine-Glycine	1.71 ± 0.18 ^b^	5.97 ± 1.11 ^a^	6.41 ± 1.95 ^a^
2	Thymidine	1.27 ± 0.13 ^b^	3.50 ± 0.19 ^a^	3.21 ± 0.32 ^a^
3	Biphenyl	0.59 ± 0.04 ^c^	1.97 ± 0.12 ^a^	1.64 ± 0.16 ^b^
4	5-Methoxytryptamine	38.13 ± 3.79 ^b^	82.28 ± 7.21 ^a^	83.54 ±7.26 ^a^
5	Glucoheptulose	3.90 ± 0.51 ^c^	7.18± 0.61 ^b^	8.02± 0.44 ^a^
6	Benzoic Acid	2.45 ± 0.20 ^c^	4.08 ±0.39 ^a^	3.44 ± 0.33 ^b^
7	Udp-Glucuronic Acid	0.32 ± 0.05 ^c^	0.44 ± 0.03 ^b^	0.53 ± 0.06 ^a^
8	Xylonolactone	1.28 ± 0.14 ^c^	1.67 ± 0.08 ^b^	2.08 ±0.17 ^a^
9	2-Monopalmitin	1.83 ± 0.14 ^c^	2.71 ± 0.21 ^b^	3.07 ± 0.32 ^a^
10	Hexadecylglycerol	3.87 ± 0.40 ^b^	6.66 ±0.58 ^a^	6.23 ± 0.77 ^a^
11	Myo-Inositol	15.64 ± 1.43 ^c^	27.05± 2.25 ^a^	23.04 ± 2.22 ^b^
12	Metanephrine	0.79 ± 0.05 ^c^	1.49 ± 0.20 ^b^	1.65 ± 0.13 ^a^
13	Cytidine	18.39 ± 1.39 ^a^	0.60 ± 0.02 ^c^	0.76 ± 0.10 ^b^
14	Diclofenac	130.89 ± 12.65 ^a^	5.58 ± 0.32 ^b^	5.80 ± 0.51 ^b^
15	Proline	9.36 ± 0.73 ^a^	2.01 ± 0.15 ^c^	2.99 ± 0.24 ^b^
16	Ethanolamine	15.57 ± 1.28 ^a^	4.31 ± 0.38 ^c^	7.46 ± 0.58 ^b^
17	Glutamate	0.37 ± 0.04 ^a^	0.14 ± 0.02 ^b^	0.15 ± 0.02 ^b^
18	Serine	2.25 ± 0.03 ^a^	0.61 ± 0.03 ^c^	1.04 ± 0.16 ^b^

D, CF and OF represented decline diseased trees, compound fertilizer treatment, bio-organic fertilizer treatment. Different lowercase letters within the same line indicate significant differences (*p* < 0.05). Boldface and normal type indicate a significant increase or decrease compared to the control, respectively.

**Table 6:** The relative contents of metabolites in rhizosphere soil of diseased bayberry trees was changed by compound fertilizer but not bio-organic fertilizer.

Metabolite Name	Relative Content	Metabolite Name	Relative Content
2-Ketoglucose imethylacetal		N-Methylalanine	
D	0.16 ± 0.05	D	2.55 ± 0.26
CF	0.30 * ±0.01	CF	1.47 ^#^ ±0.14
2-Deoxytetronic Acid		Lysine	
D	2.52 ± 0.23	D	0.54 ± 0.10
CF	4.55 * ± 0.45	CF	0.37 ^#^ ± 0.03
Galactinol		Tyrosine	
D	1.18 ± 0.10	D	1.91 ± 0.12
CF	2.88 * ± 0.21	CF	1.04 ^#^ ±0.09
Digitoxose		N-Acetyl-5-Hydroxytryptamine	
D	3.30± 0.29	D	1.42 ± 0.15
CF	4.76 * ± 0.34	CF	0.83 ^#^ ± 0.06
2,4-Diaminobutyric Acid		Montanic Acid	
D	0.93 ± 0.09	D	3.21 ± 0.47
CF	0.66 ^#^ ± 0.05	CF	1.30 ^#^ ± 0.15
Coniferin		Aconitic Acid	
D	8.53 ± 0.84	D	2.68 ± 0.18
CF	0.25 ^#^ ± 0.03	CF	1.30 ^#^ ± 0.15
Citrulline			
D	5.24 ± 0.46		
CF	2.97 ^#^ ± 0.25		

D and CF represented diseased tree alone or in combination with compound fertilizer, respectively. “*” and “^#^” represented significantly higher or lower than those of the diseased trees (*p* < 0.05).

**Table 7:** The relative contents of metabolites in rhizosphere soil of diseased bayberry trees was changed by bio-organic fertilizer but not compound fertilizer.

Metabolite Name	Relative Content	Metabolite Name	Relative Content
Arabitol		N-Acetyl-D-Hexosamine	
D	7.98 ± 0.66	D	0.33 ± 0.01
OF	14.56 * ± 1.80	OF	0.56 *± 0.02
Threitol		Deoxycholic Acid	
D	0.42 ± 0.04	D	0.45 ± 0.04
OF	0.57 * ± 0.05	OF	0.93 * ± 0.09
Inosine		Zymosterol	
D	0.65 ± 0.05	D	0.46 ± 0.03
OF	3.19 * ±0.30	OF	0.89 * ± 0.05
Glycerol		Threonic Acid	
D	195.86 ± 18.98	D	0.86 ± 0.04
OF	264.19 * ± 21.08	OF	1.34 * ± 0.14
Oleic Acid		Guanidinosuccinate	
D	1.97 ± 0.02	D	0.36 ± 0.02
OF	3.18 * ± 0.02	OF	0.78 * ± 0.08
Palmitic Acid		2’-Deoxyguanosine	
D	10.39 ± 1.02	D	0.25± 0.02
OF	18.20 * ± 1.48	OF	0.46 * ±0.05
Dodecanol		Citraconic Acid	
D	1.87 ± 0.12	D	5.12 ± 0.41
OF	4.71 * ± 0.36	OF	7.57 * ± 0.72
Thymine		Vanillic Acid	
D	1.46 ± 0.12	D	3.69 ± 0.33
OF	3.47 * ± 0.36	OF	5.78 * ± 0.65
2-Hydroxyvaleric Acid		Glucosamine	
D	2.79 ± 0.26	D	0.70 ± 0.08
OF	3.54 * ± 0.29	OF	1.04 * ± 0.06
Malonic Acid		Phthalic Acid	
D	0.21 ± 0.03	D	1.70 ± 0.20
OF	0.26 * ± 0.02	OF	2.24 * ± 0.20
Xylose		Phytanic Acid	
D	0.79 ± 0.08	D	0.58 ± 0.05
OF	1.10 * ± 0.13	OF	1.08 * ± 0.13
Glucose-1-Phosphate		Inulotriose	
D	0.35 ± 0.01	D	0.56 ± 0.05
OF	0.56 * ± 0.03	OF	0.80 * ± 0.01
Adipic Acid		Inositol-4-Monophosphate	
D	1.42 ± 0.15	D	4.81 ± 0.40
OF	2.12 * ± 0.15	OF	6.40 * ± 0.49
Glutaric Acid			
D	2.56 ± 0.16		
OF	3.67 * ± 0.29		

D and OF represented diseased tree alone or in combination with bio-organic fertilizer, respectively. “*” represented significantly higher than those of the diseased trees (*p* < 0.05).

## Data Availability

Accession to cite for these Sequence Read Archive metadata: SRP313764 and SRP313747 in https://submit.ncbi.nlm.nih.gov/subs/.

## Supplementary Materials

Supplementary data to this article can be found online at https://www.mdpi.com/article/10.3390/plants10112386/s1, Figure S1: Relative abundance of bacteria (A) and fungi (B) at the phylum level; Figure S2: Relative abundance of bacteria (A) and fungi (B) at the order level; Figure S3: Redundancy discriminant analysis (RDA) of the rhizosphere bacterial (A) and fungal (B) communities composition at phylum levels with soil physicochemical properties; Figure S4: Heatmap of correlation analysis between the microorganism relative abundances at phylum level and the metabolite relative contents of the compound fertilizer treatment; Figure S5: Heatmap of correlation analysis between the microorganism relative abundances at order level and the metabolite relative contents of the compound fertilizer treatment; Figure S6: Heatmap of correlation analysis between the microorganism relative abundances at phylum level and the metabolite relative contents of the bio-organic fertilizer treatment; Figure S7: Heatmap of correlation analysis between the microorganism relative abundances at order level and the metabolite relative contents of the bio-organic fertilizer treatment; Table S1: Contribution of soil environment to bacteria and fungi taxa at the phylum level.

## References

[1] Ren H.Y., Yu H.Y., Zhang S.W., Liang S.M., Zheng X.L., Zhang S.J., Yao P., Zheng H.K., Qi X.J. (2019). Genome sequencing provides insights into the evolution and antioxidant activity of Chinese bayberry. BMC Genom..

[2] Sun C., Huang H.Z., Xu C.J., Li X., Chen K.S. (2013). Biological activities of extracts from Chinese bayberry (*Myrica rubra* Sieb. et Zucc.): A review. Plant Foods Hum. Nutr..

[3] Ren H.Y., Wang H.Y., Qi X.J., Yu Z.P., Zheng X.L., Zhang S.W., Wang Z.S., Zhang M.C., Ahmed T., Li B. (2021). The damage caused by decline disease in bayberry plants through changes in soil properties, rhizosphere microbial community structure and metabolites. Plants.

[4] Ren H.Y., Wu C.W., Liang S.M., Wang K.Q., Zhang S.W., Zheng X.L., Qi X.J. (2020). The symptoms and nutritional status of decline diseased bayberry. Zhejiang Agric. Sci..

[5] Blanco-Canqui H., Schlegel A.J. (2013). Implications of inorganic fertilizer application of irrigated corn on soil properties: Lessons learned after 50 years. J. Environ. Qual..

[6] Shang L.R., Wan L.Q., Zhou X.X., Li S., Li X.L. (2020). Effects of organic fertilizer on soil nutrient status, enzyme activity, and bacterial community diversity in *Leymus chinensis* steppe in Inner Mongolia, China. PLoS ONE.

[7] Zubair M., Wang S.Q., Zhang P.Y., Ye J.P., Liang J.S., Nabi M., Zhou Z.Y., Tao X., Chen N., Sun K. (2020). Biological nutrient removal and recovery from solid and liquid livestock manure: Recent advance and perspective. Bioresour. Technol..

[8] Wang L., Li J., Yang F., Yaoyao E., Raza W., Huang Q., Shen Q. (2017). Application of bioorganic fertilizer significantly increased apple yields and shaped bacterial community structure in orchard soil. Microb. Ecol..

[9] Li C., Yan K., Tang L., Jia Z., Li Y. (2014). Change in deep soil microbial communities due to long-term fertilization. Soil Biol. Biochem..

[10] Yu W., Guibing Z., Liyan S., Shanyun W., Chengqing Y. (2014). Manure fertilization alters the population of ammonia-oxidizing bacteria rather than ammonia-oxidizing archaea in a paddy soil. J. Basic Microbiol..

[11] Wang Z.T., Geng Y.B., Liang T. (2020). Optimization of reduced chemical fertilizer use in tea gardens based on the assessment of related environmental and economic benefits. Sci. Total Environ..

[12] Wu L.N., Jiang Y., Zhao F.Y., He X.F., Liu H.F., Yu K. (2020). Increased organic fertilizer application and reduced chemical fertilizer application affect the soil properties and bacterial communities of grape rhizosphere soil. Sci. Rep..

[13] Zhao S., Liu D.Y., Ling N., Chen F.D., Fang W.M., Shen Q.R. (2014). Bio-organic fertilizer application significantly reduces the *Fusarium oxysporum* population and alters the composition of fungi communities of watermelon *Fusarium* wilt rhizosphere soil. Biol. Fert. Soils.

[14] Zhang H., Hua Z.W., Liang W.Z., Niu Q.H., Wang X. (2020). The prevention of bio-organic fertilizer fermented from cow manure compost by *Bacillus* sp. XG-1 on watermelon continuous cropping barrier. Int. J. Environ. Res. Public Health.

[15] Huang N., Wang W., Yao Y., Zhu F., Wang W., Chang X. (2017). The influence of different concentrations of bio-organic fertilizer on cucumber *Fusarium* wilt and soil microflora alterations. PLoS ONE.

[16] Tao C.Y., Li R., Xiong W., Shen Z.Z., Liu S.S., Wang B.B., Ruan Y.Z., Geisen S., Shen Q.R., Kowalchuk G.A. (2020). Bio-organic fertilizers stimulate indigenous soil *Pseudomonas* populations to enhance plant disease suppression. Microbiome.

[17] Tang H.M., Xiao X.P., Xu Y.L., Li C., Cheng K.K., Pan X.C., Li W. (2019). Utilization of carbon sources in the rice rhizosphere and nonrhizosphere soils with different long-term fertilization management. J. Basic Microbiol..

[18] Lucie M., Jakub R., Marketa P., Tomas M., Ondrej U. (2016). Effects of secondary plant metabolites on microbial populations: Changes in community structure and metabolic activity in contaminated environments. Int. J. Mol. Sci..

[19] Wang Y.C., Wu B.D., Jiang K. (2019). Allelopathic effects of Canada goldenrod leaf extracts on the seed germination and seedling growth of lettuce reinforced under salt stress. Ecotoxicology.

[20] Zhou X.A., Wu F.Z. (2012). P-Coumaric acid influenced cucumber rhizosphere soil microbial communities and the growth of *Fusarium oxysporum* f.sp. cucumerinum Owen. PLoS ONE.

[21] Zhang Y., Tao Y., Zhang H., Wang L., Sun G.Q., Sun X., Erinle K.O., Feng C.C., Song Q.X., Li M. (2015). Effect of di-n-butyl phthalate on root physiology and rhizosphere microbial community of cucumber seedings. J. Hazard. Mater..

[22] Zhao L.J., Zhang H.L., Jason C.W., Chen X.Q., Li H.B., Qu X.L., Rong J. (2019). Metabolomics reveals that engineered nanomaterial exposure in soil alters both soil rhizosphere metabolite profiles and maize metabolic pathways. Environ. Sci. Nano.

[23] Tian L.Y., Shen J.P., Sun G.X., Wang B., Ji R., Zhao L.J. (2020). Foliar application of SiO_2_ nanoparticles alters soil metabolite profiles and microbial community composition in the pakchoi (*Brassica chinensis* L.) rhizosphere grown in contaminated mine soil. Environ. Sci. Technol..

[24] Ghimire B.K., Ghimire B., Yu C.Y., Chung I.M. (2019). Allelopathic and autotoxic effects of medicago sativa-derived allelochemicals. Plants.

[25] Pan L., Lei D.Y., Jin L., He Y., Yang Q.Q. (2018). Promising fungicides from allelochemicals: Synthesis of umbelliferone derivatives and their structure-activity relationships. Molecules.

[26] Shibl A.A., Isaac A., Ochsenkuhn M.A., Cardenas A., Fei C., Behringerc G., Arnoux M., Drou N., Santos M.P., Gunsalus K.C. (2020). Diatom modulation of select bacteria through use of two unique secondary metabolites. Proc. Natl. Acad. Sci. USA.

[27] Neal A.L., Ahmad S., Gordon-Weeks R., Ton J. (2012). Benzoxazinoids in root exudates of maize attract *Pseudomonas putida* to the rhizosphere. PLoS ONE.

[28] Sogi D.S., Siddiq M., Dolan K.D. (2015). Total phenolics, carotenoids and antioxidant properties of tommy atkin mango cubes as affected by drying techniques. LWT Food Sci. Technol..

[29] Jiang Y., Nie W.J. (2015). Chemical properties in fruits of mulberry species from the Xinjiang province of China. Food Chem..

[30] Qu B.P., Liu Y.X., Sun X.Y., Li S.Y., Wang S.Y., Xiong K.Y., Yun B.H., Zhang H. (2019). Effect of various mulches on soil physico-chemical properties and tree growth (*Sophora japonica*) in urban tree pits. PLoS ONE.

[31] Ma M.C., Zhou J., Ongena M., Liu W.Z., Wei D., Zhao B., Guan D.W., Jiang X., Li J. (2018). Effect of long-term fertilization strategies on bacterial community composition in a 35-year field experiment of Chinese mollisols. AMB Express.

[32] Baran A., Mierzwa-Hersztek M., Gondek K., Tarnawski M., Szara M., Gorczyca O., Koniarz T. (2019). The influence of the quantity and quality of sediment organic matter on the potential mobility and toxicity of trace elements in bottom sediment. Environ. Geochem. Health.

[33] Wu L.Y., Wen C.Q., Qin Y.J., Yin H.Q., Tu Q.C., Nostrand J.D.V., Yuan T., Yuan M.T., Deng Y., Zhou J.Z. (2015). Phasing amplicon sequencing on Illumina Miseq for robust environmental microbial community analysis. BMC Microbiol..

[34] Mukherjee P.K., Chandra J., Retuerto M., Sikaroodi M., Brown R.E., Jurevic R., Salata R.A., Lederman M.M., Gillevet P.M., Ghannoum M.A. (2014). Oral mycobiome analysis of HIV-infected patients: Identification of pichia as an antagonist of opportunistic fungi. PLoS Pathog..

[35] Bolger A.M., Lohse M., Usadel B. (2014). Trimmomatic: A flexible trimmer for Illumina *Caporaso* sequence data. Bioinformatics.

[36] Reyon D., Tsai S.Q., Khayter C., Foden J.A., Sander J.D., Joung J.K. (2012). FLASH assembly of TALENs for high-throughput genome editing. Nat. Biotech..

[37] Rognes T., Flouri T., Nichols B., Quince C., Mahé F. (2016). VSEARCH: A versatile open source tool for metagenomics. PeerJ.

[38] Caporaso J.G., Kuczynski J., Stombaugh J., Bittinger K., Bushman F.D., Costello E.K., Fierer N., Peña A.G., Goodrich J.K., Gordon J.I. (2010). QIIME allows analysis of high-throughput community sequencing data. Nat. Methods.

[39] Wang Q., Garrity G.M., Tiedje J.M., Cole J.R. (2007). Naive Bayesian classifier for rapid assignment of rRNA sequences into the new bacterial taxonomy. Appl. Environ. Microb..

[40] Johnson M., Zaretskaya I., Raytselis Y., Merezhuk Y., McGinnis S., Madden T.L. (2008). NCBI BLAST: A better web interface. Nucleic Acids Res..

[41] Chao J., Bunge J. (2002). Estimatin the number of species in a stochastic abundance model. Biometrics.

[42] Hill T.C., Walsh K.A., Harris J.A., Moffett B.F. (2003). Using ecological diversity measures with bacterial communities. FEMS Microbiol. Ecol..

[43] Li X.P., Liu C.L., Zhao H., Gao F., Ji G.N., Hu F., Li H.X. (2018). Consistent improvements in soil biochemical properties and crop yields by organic fertilization for above-ground (rapeseed) and below-ground (sweet potato) crops. J. Agric. Sci..

[44] Lin W.W., Lin M.H., Zhou H.Y., Wu H.M., Li Z.W., Lin W.X. (2019). The effects of chemical and organic fertilizer usage on rhizosphere soil in tea orchards. PLoS ONE.

[45] Jiang S.Q., Yu Y.N., Gao R.W., Wang H., Zhang J., Li R., Long X.H., Shen Q.R., Chen W., Cai F. (2019). High-throughput absolute quantification sequencing reveals the effect of different fertilizer applications on bacterial community in a tomato cultivated coastal saline soil. Sci. Total Environ..

[46] Liu Z., Guo Q., Feng Z.Y., Liu Z.D., Li H.Y., Sun Y.F., Liu C.S., Lai H.X. (2020). Long-term organic fertilization improves the productivity of kiwifruit (*Actinidia chinensis* Planch.) through increasing rhizosphere microbial diversity and network complexity. Appl. Soil Ecol..

[47] Qiao C.C., Penton C.R., Xiong W., Liu C., Wang R.F., Liu Z.Y., Xu X., Li R., Shen Q. (2019). Reshaping the rhizosphere microbiome by bio-organic amendment to enhance crop yield in a maize-cabbage rotation system. Appl. Soil Ecol..

[48] Jiang X.M., Zhang F.H., Li J.H., Fan H., Cheng Z.B., Wang K.Y. (2016). Effects of bio-organic fertilizer on soil microbiome against *Verticillium dahliae*. Int. J. Agric. Biol..

[49] Gao M., Zhou J.J., Wang E.T., Chen Q., Xu J., Sun J.G. (2015). Multiphasic characterization of a plant growth promoting bacterial strain, *Burkholderia* sp 7016 and its effect on tomato growth in the field. J. Int. Agric..

[50] Maqsood A., Shahid M., Hussain S., Mahmood F., Basit F. (2020). Root colonizing *Burkholderia* sp. AQ12 enhanced rice growth and upregulated tillering-responsive genes in rice. Appl. Soil Ecol..

[51] Borrell A.N., Shi Y., Gan Y., Bainard L.D., Germida J.J., Hamel C. (2017). Fungal diversity associates with pluses and its influence on the subsequent wheat crop in the Canadian prairies. Plant Soil.

[52] Sallam N.M.A., Eraky A.M.I., Sallam A. (2019). Effect of *Trichoderma* spp. on *Fusarium* wilt disease of tomato. Mol. Biol. Rep..

[53] Li X.Q., Su Y., Ahmed T., Ren H.Y., Javed M.R., Yao Y.L., An Q.L., Yan J.L., Li B. (2021). Effects of different organic fertilizers on improving soil from newly reclaimed land to crop soil. Agriculture.

[54] Patra B., Ray S., Richter A., Majumder A.L. (2010). Enhanced salt tolerance of transgenic tobacco plants by co-expression of *PcINO1* and *McIMT1* is accompanied by increased level of myo-inositol and methylated inositol. Protoplasma.

[55] Pierattini E.C., Francini A., Huber C., Sebastiani L., Schröder P. (2018). Poplar and diclofenac pollution: A focus on physiology, oxidative stress and uptake in plant organs. Sci. Total Environ..

[56] Li R.Y., Pang Z.Q., Zhou Y.M., Fallah N., Hu C.H., Lin W.X., Yuan Z.N. (2020). Metagenomic analysis exploring taxonomic and functional diversity of soil microbial communities in sugarcane fields applied with organic fertilizer. Biomed Res. Int..

[57] Zhou D., Huang X.F., Chaparro J.M., Badri D.V., Guo J. (2015). Root and bacterial secretions regulate the interaction between plants and PGPR leading to distinct plant growth promotion effects. Plant Soil.

[58] Baccolini C., Witte C.P. (2019). AMP and GMP catabolism in *Arabidopsis* converge on xanthosine, which is degraded by a nucleoside hydrolase heterocomplex. Plant Cell.

[59] Huang X.F., Chaparro J.M., Reardon K.F., Zhang R.F., Shen Q.R., Vivanco J.M. (2014). Rhizosphere interactions: Root exudates, microbes, and microbial communities. Botany.

[60] Jiang J., Yuan X., Huang G., Shi W., Yang X., Jiang Q., Jia Y., Yang X., Jiang H. (2020). Hepatopancreatic metabolomics shedding light on the mechanism underlying unsynchronized growth in giant freshwater prawn, *Macrobrachium rosenbergii*. PLoS ONE.

[61] Adams Z.P., Ehlting J., Edwards R. (2019). The regulatory role of shikimate in plant phenylalanine metabolism. J. Theor. Biol..

[62] Wong-Ng J., Celani A., Vergassola M. (2018). Exploring the function of bacterial chemotaxis. Curr. Opin. Microbiol..

[63] Cui K., Zhao Y., He L., Ding J., Li B., Mu W., Liu F. (2020). Comparison of transcriptome profiles of the fungus *Botrytis cinerea* and insect pest *Bradysia odoriphaga* in response to benzothiazole. Front. Microbiol..

[64] Westhoff S., Otto S.B., Swinkels A., Bode B., van Wezel G.P., Rozen D.E. (2020). Spatial structure increases the benefits of antibiotic production in Streptomyces. Evolution.

[65] Coelho A.I., Berry G.T., Rubio-Gozalbo M.E. (2015). Galactose metabolism and health. Curr. Opin. Clin. Nutr. Metab. Care.

[66] Tiwari K., Dubey V.K. (2018). Fresh insights into the pyrimidine metabolism in the trypanosomatids. Parasit Vectors.

