# Supplementary material for: Effect of Two Kinds of Fertilizers on Growth and Rhizosphere Soil Properties of Bayberry with Decline Disease
Source: Plants (Basel). 2021 Nov 5;10(11):2386. doi: 10.3390/plants10112386 (PMC8624721; doi:10.3390/plants10112386)
Supplement: Supplementary file 1 [file plants-10-02386-s001.zip › plants-1454610-SI.pdf]

Supplementary data

Table S1. Contribution of soil environment to bacteria and fungi taxa at the phylum level.

Soil environment	Contribution at the bacterial phylum level (%)	Contribution at the fungal phylum level (%)
pH	3.2	1.4
Organic Matter	5.6	8.6
Alkali-hydrolyzable nitrogen	14.2	0.3
Available Phosphorus	7.1	8.4
Exchangeable calcium	11.2	5.3
Exchangeable magnesium	11.2	1.1

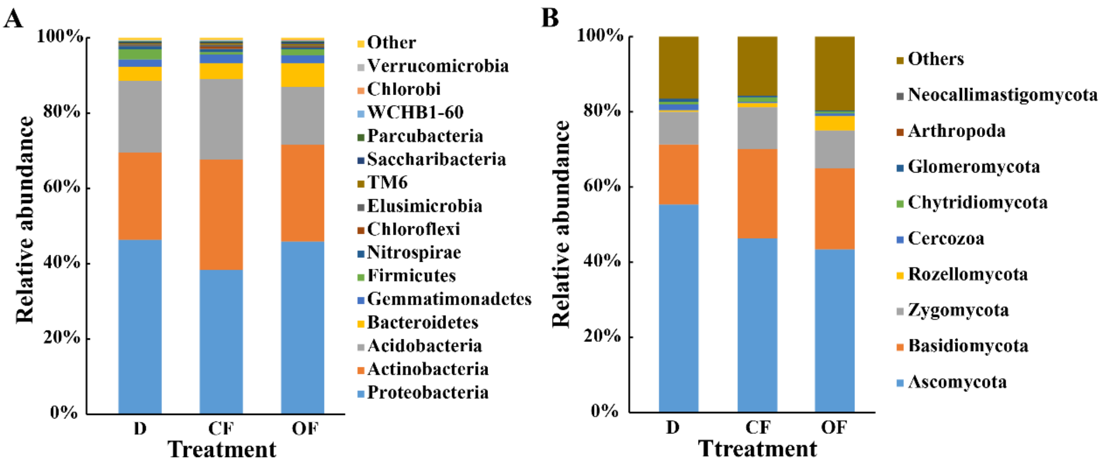

Figure S1. Relative abundance of bacteria (A) and fungi (B) at the phylum level. D, CF, and OF represented decline diseased trees, in combination with either compound fertilizer or bio-organic fertilizer, respectively.

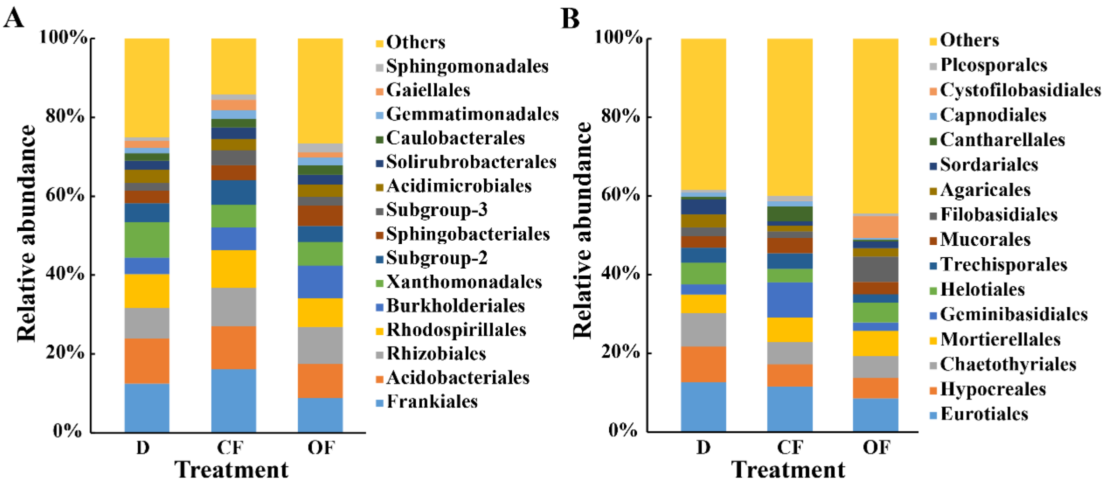

Figure S2. Relative abundance of bacteria (A) and fungi (B) at the order level. D, CF, and OF represented decline diseased trees, in combination with either compound fertilizer or bio-organic fertilizer, respectively.

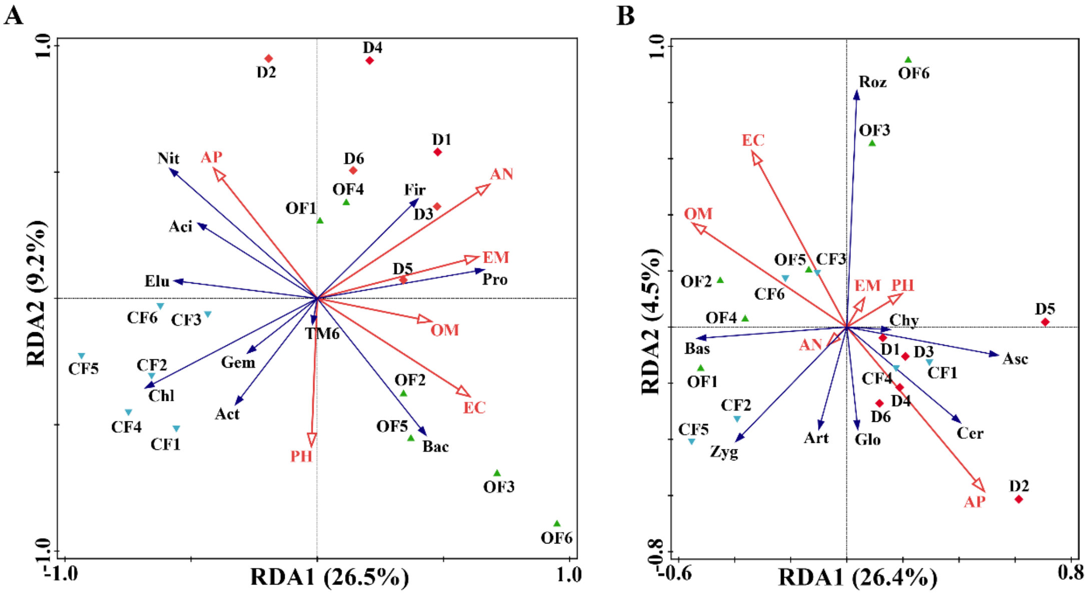

Figure S3. Redundancy discriminant analysis (RDA) of the rhizosphere bacterial (A) and fungal (B) communities composition at phylum levels with soil physicochemical properties. Pro: Proteobacteria; Act: Actinobacteria; Aci: Acidobacteria; Bac: Bacteroidetes; Gem: Gemmatimonadetes; Fir: Firmicutes; Nit: Nitrospirae; Chl: Chloroflexi; Elu: Elusimicrobia; Asc: Ascomycota; Bas: Basidiomycota; Zyg: Zygomycota; Roz: Rozellomycota; Cer: Cercozoa; Chy: Chytridiomycota; Glo: Glomeromycota; Art: Arthropoda; PH: pH; OM: organic matter; AN: alkali-hydrolyzable nitrogen; AP: available phosphorus; EC: exchangeable calcium; EM: exchangeable magnesium. Red diamond: samples from Decline bayberry; Blue down triangle: sample from compound fertilizer treated trees (CF); Green up triangle: sample from bio-organic fertilizer treated trees (OF).

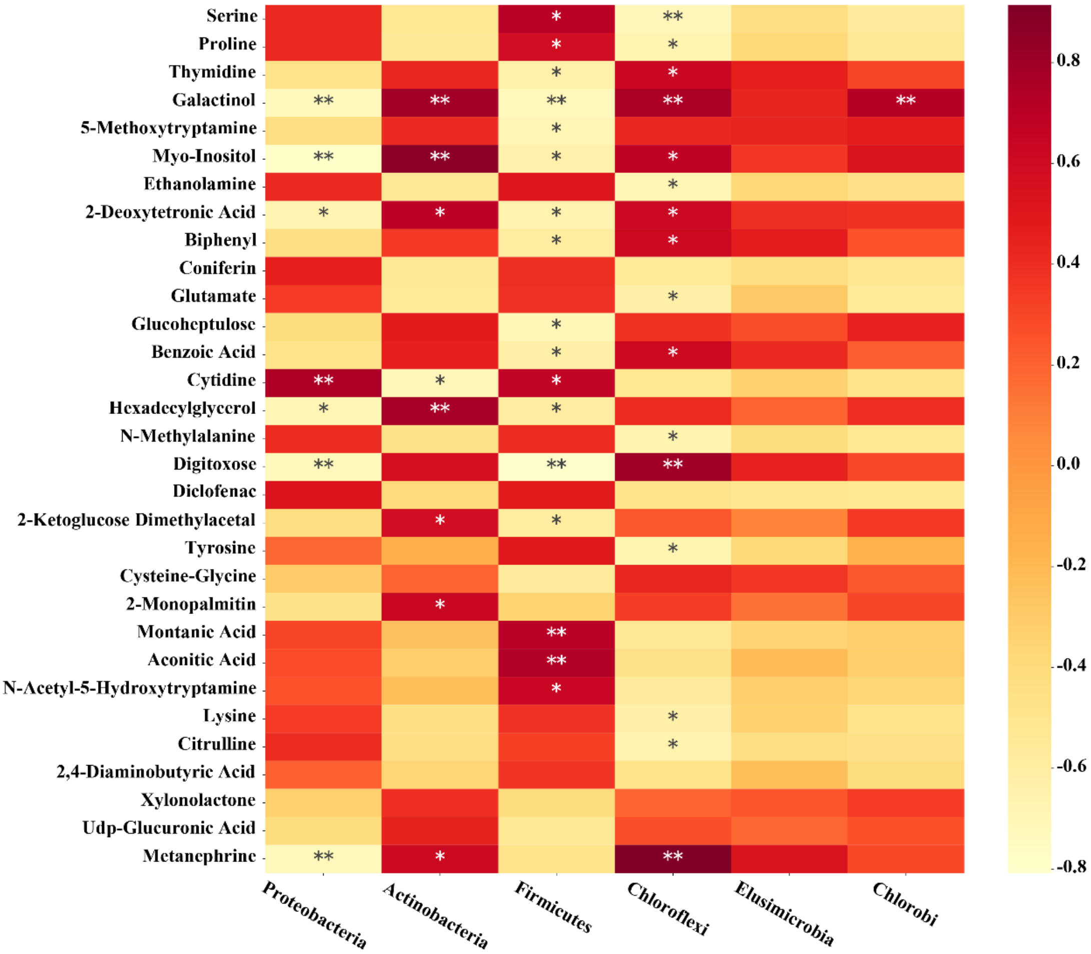

Figure S4. Heatmap of correlation analysis between the microorganism relative abundances at phylum level and the metabolite relative contents of the compound fertilizer treatment. * and ** indicate a significant correlation at $P < 0.05$ and $P < 0.01$, respectively. The depth of the orange scale represents the magnitude of the correlation coefficient, the darker color is the greater positive correlation, the lighter color is the greater negative correlation.

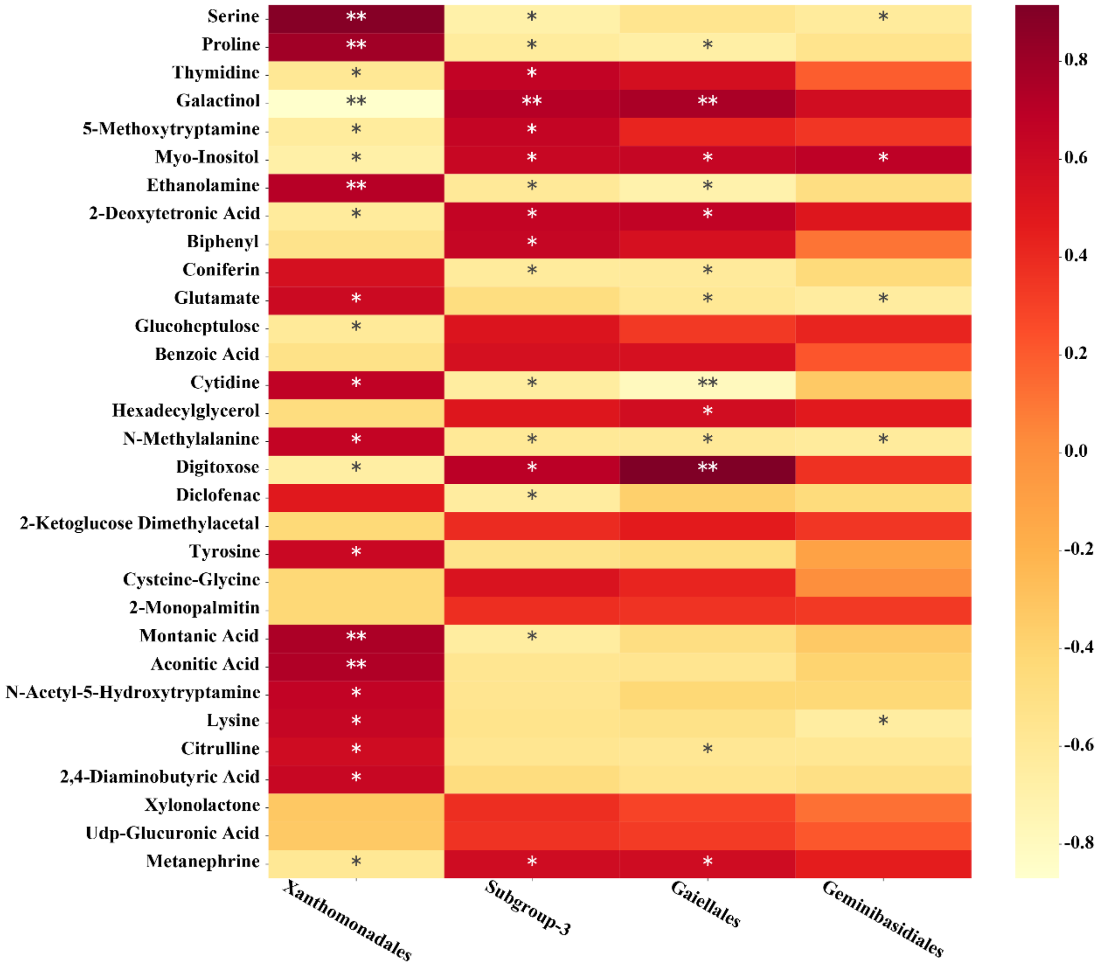

Figure S5. Heatmap of correlation analysis between the microorganism relative abundances at order level and the metabolite relative contents of the compound fertilizer treatment. * and ** indicate a significant correlation at $P < 0.05$ and $P < 0.01$, respectively. The depth of the orange scale represents the magnitude of the correlation coefficient, the darker color is the greater positive correlation, the lighter color is the greater negative correlation.

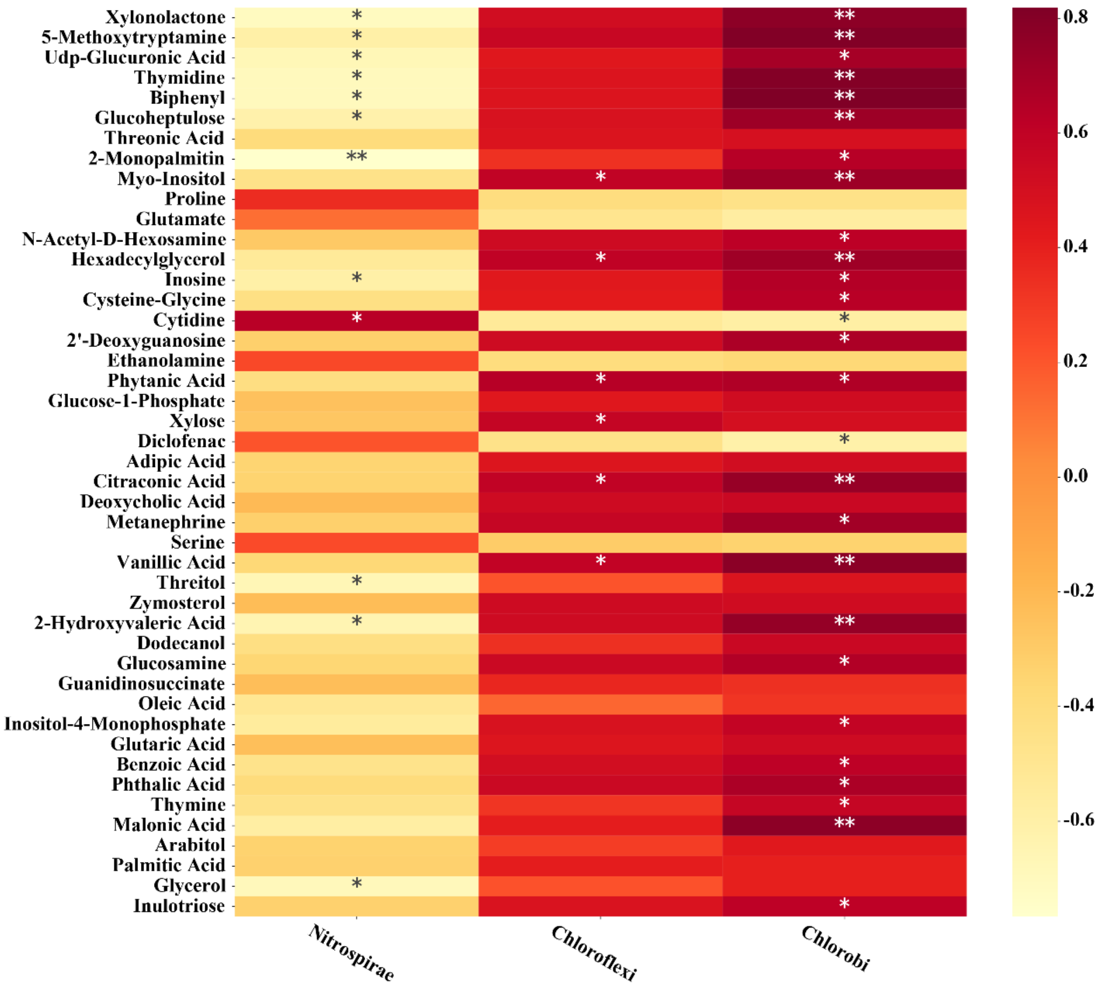

Figure S6. Heatmap of correlation analysis between the microorganism relative abundances at phylum level and the metabolite relative contents of the bio-organic fertilizer treatment. * and ** indicate a significant correlation at $P < 0.05$ and $P < 0.01$, respectively. The depth of the orange scale represents the magnitude of the correlation coefficient, the darker color is the greater positive correlation, the lighter color is the greater negative correlation.

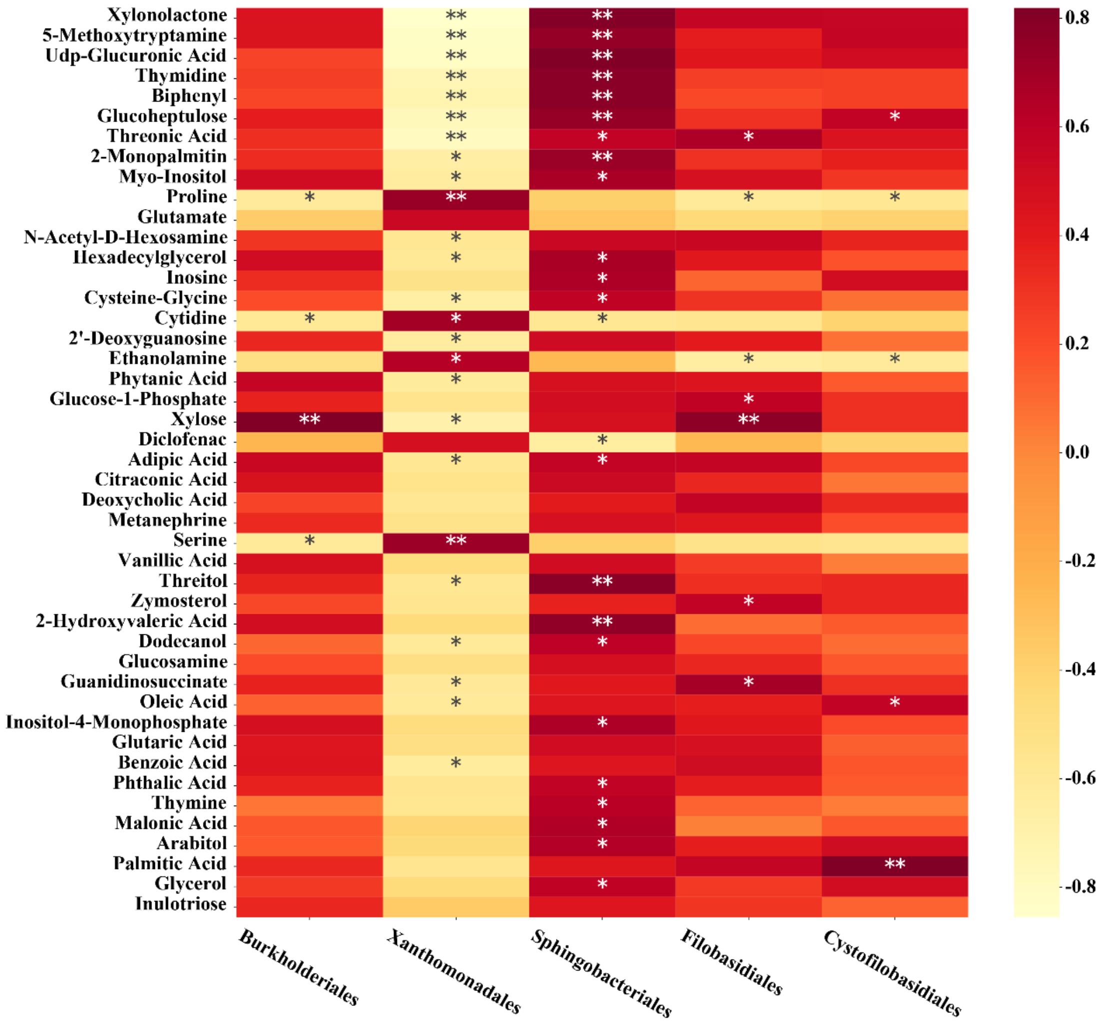

Figure S7. Heatmap of correlation analysis between the microorganism relative abundances at order level and the metabolite relative contents of the bio-organic fertilizer treatment. * and ** indicate a significant correlation at $P < 0.05$ and $P < 0.01$, respectively. The depth of the orange scale represents the magnitude of the correlation coefficient, the darker color is the greater positive correlation, the lighter color is the greater negative correlation.
